# A potential third Manta Ray species near the Yucatán Peninsula? Evidence for a recently diverged and novel genetic *Manta* group from the Gulf of Mexico

**DOI:** 10.7717/peerj.2586

**Published:** 2016-11-01

**Authors:** Silvia Hinojosa-Alvarez, Ryan P. Walter, Pindaro Diaz-Jaimes, Felipe Galván-Magaña, E. Misty Paig-Tran

**Affiliations:** 1 Posgrado en Ciencias del Mar y Limnología/Laboratorio de Genética de Organismos Acuáticos, Instituto de Ciencias del Mar y Limnología, Universidad Nacional Autónoma de México, Ciudad de México, Mexico; 2 Instituto de Biotecnología, Chamilpa, Universidad Nacional Autónoma de México, Cuernavaca, Morelos, México; 3 Department of Biological Science, California State University, Fullerton, CA, United States; 4 Instituto de Ciencias del Mar y Limnología, Unidad Académica de Ecología y Biodiversidad Acuática, Laboratorio de Genética de Organismos Acuáticos, Universidad Nacional Autónoma de México, Ciudad de México, Mexico; 5 Instituto Politécnico Nacional, Centro Interdisciplinario de Ciencias Marinas, La Paz, Baja California Sur, México

**Keywords:** *Manta alfredi*, Yucatán manta ray, ND5, Genetic divergence, *Manta birostris*

## Abstract

We present genetic and morphometric support for a third, distinct, and recently diverged group of Manta ray that appears resident to the Yucatán coastal waters of the Gulf of Mexico. Individuals of the genus *Manta* from Isla Holbox are markedly different from the other described manta rays in their morphology, habitat preference, and genetic makeup. Herein referred to as the Yucatán Manta Ray, these individuals form two genetically distinct groups: (1) a group of mtDNA haplotypes divergent (0.78%) from the currently recognized *Manta birostris* and *M. alfredi* species, and (2) a group possessing mtDNA haplotypes of *M. birostris* and highly similar haplotypes. The latter suggests the potential for either introgressive hybridization between Yucatán Manta Rays and *M. birostris*, or the retention of ancestral *M. birostris* signatures among Yucatán Manta Rays. Divergence of the genetically distinct Yucatán Manta Ray from *M. birostris* appears quite recent (<100,000 YBP) following fit to an Isolation-with-Migration model, with additional support for asymmetrical gene flow from *M. birostris* into the Yucatán Manta Ray. Formal naming of the Yucatán Manta Ray cannot yet be assigned until an in-depth taxonomic study and further confirmation of the genetic identity of existing type specimens has been performed.

## Introduction

The genus *Manta* currently comprises two recently re-described species, *Manta alfredi* (Krefft, 1868) and *M. birostris* (Walbaum, 1792), that occur circumglobally in tropical and subtropical seas (Marshall, Compagno & Bennett, 2009). *Manta alfredi* (Reef Manta Ray) is distinguished from *M. birostris* (Giant Manta Ray) by habitat preference, a smaller overall disc width, unique color morphology, and the absence of a vestigial spine (Marshall, Compagno & Bennett, 2009); however, these characteristics are not mutually distinct and reliable identification of these species remains problematic (Kashiwagi et al., 2012).

Recent genetic evidence supports Marshall, Compagno & Bennett (2009) morphological and ecological separation of *M. birostris* and *M. alfredi* (Kashiwagi et al., 2012). Using the mitochondrial gene *ND5*, Kashiwagi et al. (2012) estimated a recent divergence time of 0.275-1MYA for these species. Interestingly, Kashiwagi et al.’s (2012) data also inadvertently demonstrated that field identification of mantas based on morphology is unreliable. For example, several specimens visually identified in the field as *M. alfredi* (iv and v in Kashiwagi et al., 2012), separated out genetically as *M. birostris*. These anomalies indicate that visual identification is not as accurate as Marshall, Compagno & Bennett (2009) suggest. Adding to this confusion, Walter et al. (2014) confirmed the existence of a hybrid manta specimen based on heterozygous *RAG1* polymorphisms that was visually identified as *M. alfredi* in the field based on the Marshall, Compagno & Bennett (2009) guide. Using visual identification as the sole method for identification of *Manta* spp. is further complicated by their ability to display rapid (within minutes), reversible changes to their external color morphology, especially along the dorsal surface (Ari, 2014).

Using visual identification to separate manta species is problematic and depends greatly on the parameters one uses to define a species. For example, the hybridization of *M. birostris* and *M. alfredi* indicates that the genus *Manta* cannot be separated into two distinctive groups of non-interbreeding biological “species.” Furthermore, the overlap in similar anatomical traits—leading to visual misidentifications—does not conform to strict morphological species parameters. We note that the concept of what comprises a species is fluid and can be defined in many different ways. Marshall, Compagno & Bennett (2009) alluded to a third possible putative species that is endemic to the Caribbean Sea, based on differences in morphology and coloration patterns. This third species is strongly associated with a highly productive area of the Gulf Stream Current (Graham et al., 2012). A population of manta rays near Isla Holbox, situated off the Yucatán Peninsula, fits this description as they (1) feed in the area and (2) appear to be secluded to the Caribbean by the Gulf Stream Current. Adding further confusion to the manta lineage, this resident population of manta rays present off the coast of the Yucatán Peninsula displays morphological similarities to both *M. birostris* and *M. alfredi* (Marshall, Compagno & Bennett, 2009; Couturier et al., 2012; Graham et al., 2012; Poortvliet et al., 2015). It is unclear whether this is truly a third putative species or simply an isolated breeding population of *M. birostris*.

Our goals for this paper are to determine if molecular data and spatial distribution support the classification of a third, distinctive manta species; herein referred to as the Yucatán Manta Ray.

## Methods

### Study Area

**Figure 1:**
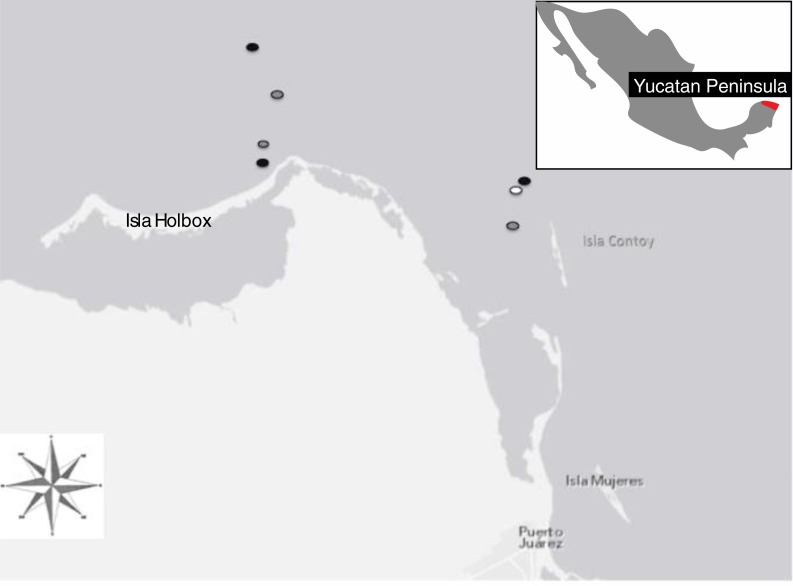
Location of sampling sites located at the northernmost tip of the Yucatán peninsula (21°31′18″N and 87°22′36″W) including the locations of manta rays surveyed via boat in 2010 (black dots), aerial surveys 2010 (white dots), and boat in 2011(grey dots). Mantas were reliably found in Marine Protected Areas near Isla Holbox and Isla Contoy.

Isla Holbox is located off the Northeast tip of the Yucatán Peninsula Mexico, in the Yum Balam marine protected area (Fig. 1). It is located between 21°43′ and 21°14′ latitude, and between 87°32′ and 87°07′ longitude. The area is characterized by endemic, rare and endangered species such as the Mexican crocodile, manatees, dolphins, turtles, whale sharks and seabirds. The waters off the Yucatán Peninsula are strongly influenced by the Yucatán channel current resulting in seasonal upwelling events, which occur from May to September (Hinojosa-Alvarez, 2009). During these upwelling events, cold, nutrient enriched waters support an increase in plankton production known locally as “surgencia de Cabo Catoche” (Zavala-Hidalgo, Morey & O’brien, 2003). This increase in plankton biomass results in seasonal congregations of large filter-feeding elasmobranchs including the Whale shark, *Rhincodon typus*, and resident Manta rays.

### Environmental Data and Morphology

Manta rays in the Yucatán Peninsula were located feeding at the surface in plankton-rich upwelling areas between 21°46.020′N and 87°01.200′W and 21°30.00′ and 86°4100 (Fig. 1).

**Figure 2:**
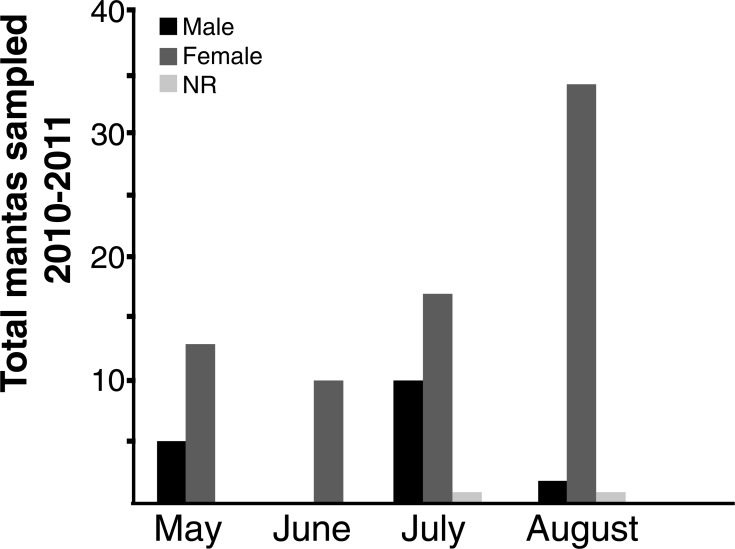
Manta Ray total abundance during “Manta season.” NR, no sex recorded.

Videos of Yucatán Manta Rays were taken over two consecutive years in 2010 and 2011, during prime “manta season” from May through August, when mantas were seen feeding at the ocean surface. Each individual was sexed via visual confirmation of the presence or absence of claspers and essential species identification data were documented (e.g., dorsal and ventral coloration patterns, presence or absence of a caudal spine, mouth coloration and presence or absence of dark spots between the fifth gill slits). The number and sex of manta rays were recorded each month for both years (2010 & 2011) to assess how population dynamics change during the span of a full “manta season” (Fig. 2).

To prevent resampling of specimens, we edited the videos using Quick Time Player and Adobe Photoshop CS5 Master Collection and obtained ventral photographs of each individual (Supplemental Information 1). Then we compared each photograph using the Individual Identification System I3S (Pierce 2007) available on the Internet (http://www.reijns.com/i3s/).

Each month during “manta season” we downloaded satellite images of chlorophyll-*α* concentrations (NOAA satellite Modis—Aua) to estimate the abundance of available nutrients in areas where we visually confirmed the presence of manta rays.

### Genetics

Tissue samples for DNA isolation were collected using a modified pole spear and stored in 10 ml of salt saturated DMSO (Dimethyl sulfoxide). Tissue samples were small (less than 1 cm) and mantas did not experience lasting effects from these relatively small biopsy samples. All fieldwork was conducted with knowledge and permission from the Comision Nacional de Areas Naturales Protegidas (CONANP), Mexico.

DNA was extracted from tissue samples using the Wizard Genomic DNA Purification kit (Promega) following manufacturer’s instructions. The polymerase chain reaction (PCR) was used to amplify an 1,188 bp fragment of the mitochondrial *ND5* region using primers MLF2 (5′-TGGTGCAACTCCAAGCTAAA-3′) and MNR4 (5′-TCAGGCGTTR AGGTATGATG-3′) as described by Kashiwagi et al. (2012) from a total of 12 tissue samples from Yucatán Manta Rays. We also amplified an approximately 750 bp fragment of the nuclear *RAG1* gene using the primers MRAGF2 (5-GGGAGCAGATATTCCAACCA-3) and MRAGR2 (5-TTCTCTTCGTGGCTCCTTGT-3) also described by Kashiwagi et al. (2012) as a diagnostic marker between *Manta birostris* and *Manta alfredi*. BigDye Terminator (Applied Biosciences) sequencing reactions were performed using the above PCR primers and MRND5L1 (5′-ATCGGTTGAGAAGGTGTAGGA-3′; Clark, 2002) and MNR2 (5′-TAGGGCAGAGACTGGCGTAG-3′; Kashiwagi et al., 2012) for the *ND5* fragment, and for the *RAG1* fragment using the above suite of MRAG PCR primers. Sequence data was edited and aligned with available *ND5* and *RAG1* sequences for *M. birostris* and *M. alfredi* retrieved from NCBI Genbank (Supplemental Information 2), and trimmed to 1,067 bp and 681 bp for each DNA fragment, respectively (Table 1). Haplotypes were identified in DnaSP 5.0 (Librado & Rozas, 2009). Likelihood scores for 77 nucleotide models were performed on an alignment of all known *ND5 Manta* haplotypes and the Yucatán samples in jModelTest 2.0 (Darriba et al., 2012).

**Table 1:** Unique Mitochondrial *ND5* haplotypes and polymorphic sites for Yucatán Manta Rays sampled for this paper, and *M. birostris* and *M. alfredi* from Kashiwagi et al. (2012) (MB01-MB12, MA01-MA05), and Walter et al. (2014) (Ma18). A dash (-) at a position indicates a nucleotide that is identical to the first listed haplotype.

	Nucleotide position																											
Haplotype/Sample	8	20	23	68	76	81	89	128	149	185	215	275	325	393	416	456	503	569	575	608	620	621	815	824	827	869	875	953	980
Yucatán Manta																													
YM03, YM04, YM07, YM08, YM12	A	C	T	T	C	**A**	**C**	C	C	A	A	A	**A**	T	A	T	T	**A**	A	T	G	C	**T**	A	G	**G**	C	G	T
YM05	-	-	-	-	-	G	T	-	-	-	-	-	G	-	G	-	-	G	-	-	-	-	C	-	-	A	-	-	C
YM06, YM10, YM11	-	-	-	-	-	G	T	-	-	-	-	-	G	-	-	-	-	G	-	-	-	-	C	-	-	A	-	-	C
YM09	-	-	-	-	-	-	-	-	-	-	-	-	-	-	-	-	-	-	-	-	-	-	-	-	-	A	T	-	-
YM13	-	-	-	-	-	G	T	-	-	-	-	-	-	-	-	-	-	G	-	-	-	-	C	-	-	A	-	-	C
YM14	-	-	-	-	-	-	-	-	-	-	-	-	G	-	-	-	-	G	-	-	-	A	C	-	-	A	-	-	C
*Manta birostris*																													
MB01	-	-	-	-	-	G	T	-	-	-	-	-	G	-	-	-	-	G	-	-	-	-	C	-	-	A	-	-	C
MB02	G	-	-	-	-	G	T	-	-	-	-	-	G	-	-	-	-	G	-	-	-	-	C	-	-	A	-	-	C
MB03	-	-	-	-	-	G	T	-	-	-	G	-	G	-	-	-	-	G	-	-	-	-	C	-	-	A	-	-	C
MB04	-	-	-	-	-	G	T	-	T	-	-	-	G	-	-	-	-	G	-	-	-	-	C	-	-	A	-	-	C
MB05	-	-	-	-	-	G	T	-	-	-	-	-	G	-	G	C	-	G	-	-	-	-	C	-	-	A	-	-	C
MB06	-	-	-	-	-	G	T	-	-	-	-	-	G	-	G	-	-	G	-	-	-	-	C	-	-	A	-	-	C
MB07	-	-	-	-	-	G	T	-	-	-	-	-	G	-	G	-	-	G	-	-	-	-	C	G	-	A	-	-	C
MB08	-	-	-	C	-	G	T	-	-	-	-	-	G	-	-	-	C	G	-	-	-	-	C	-	-	A	-	-	C
MB09	-	-	-	-	-	G	T	-	-	-	-	-	G	-	-	-	-	G	-	C	-	-	C	-	-	A	-	A	C
MB10	-	-	-	-	A	G	T	-	-	-	-	-	G	-	-	-	-	G	-	-	-	-	C	-	-	A	-	-	C
MB11	-	-	-	-	-	G	T	-	-	-	-	-	G	-	-	-	-	G	G	-	-	-	C	-	-	A	-	-	C
MB12	-	-	-	-	-	G	-	-	-	G	-	G	-	-	-	-	-	-	-	-	A	-	-	-	-	-	-	-	C
*Manta alfredi*																													
MA01	-	T	-	-	-	G	-	T	-	-	-	-	G	-	-	-	-	G	-	-	A	-	C	-	-	-	T	-	C
MA02	-	T	-	-	-	G	-	T	-	-	-	-	G	-	-	-	-	G	-	-	A	-	C	-	-	-	T	A	C
MA03	-	T	-	-	-	G	-	T	-	-	-	-	G	C	-	-	-	G	-	-	A	-	C	-	-	-	T	A	C
MA04	-	-	-	-	-	G	T	-	-	-	-	-	G	-	-	-	-	G	-	-	-	-	C	-	-	A	-	-	C
MA05	-	-	-	-	-	G	T	-	-	-	-	-	G	-	-	-	-	G	-	-	-	-	C	-	A	A	-	-	C
Ma18	-	T	C	-	-	G	-	T	-	-	-	-	G	-	-	-	-	G	-	-	A	-	C	-	-	-	T	-	C

Following Akaike Information Criteria (AIC/AICc) and Bayesian Information Criteria (BIC) analyses in jModelTest, support was most consistent for the TrN+I model (p-invar = 0.91) for downstream phylogenetic analyses. Using *Mobula japanica* as an outgroup (Accession #NC_018784), phylogenetic trees were constructed using four approaches: (1) Neighbor-joining in PAUP 4.0.b5 (Swofford, 2001); (2) Maximum parsimony in MEGA7 (Kumar, Stecher & Tamura, 2016); (3) Maximum-Likelihood in RaxML (Stamatakis, 2014); and (4) Bayesian using MrBayes 3.1.2 (Ronquist & Huelsenbeck, 2003). A statistical parsimony haplotype network was constructed in TCS (Clement, Posada & Crandall, 2000) using the 95% connection limit, to visualize haplotype sharing among samples and the genetic relationship among haplotypes.

Coalescent-based estimates of time-splitting and coalescent migration rates between Yucatán Manta Rays and *M. birostris* were performed by fitting the data to an Isolation-with-Migration model (IM) in IMa2 (Hey & Nielsen, 2004; Hey & Nielsen, 2007; Hey, 2010). IM runs were performed in triplicate M-mode runs (with different randomization seeds) using a 10 heated MCMC chains, a long burn-in period (minimum 50 × 10^6^ iterations, ESS >200), followed by the collection of 300,000 genealogies (Hey, 2010), assuming a generation time of 20 years. A final L-mode run was performed drawing 100,000 genealogies from the triplicate runs to assess the best fit of demographic models to the observed data using a likelihood ratio (LLR) test and AIC. A suite of published mutation rates (Table 2) was applied to estimate time-splitting and demographic parameters. All phylogenetic and IM analyses were also performed using a reduced individual dataset, covering a slightly longer *ND5* fragment of 1,136 bp, for Yucatán individuals aligned with Kashiwagi et al. (2012) *Manta ND5* haplotypes (see Supplemental Information 2).

**Table 2:** Comparison of various elasmobranch substitution rates for estimating the split time between Yucatán Manta Rays and *Manta birostris* following IM analysis (MLE peak = 0.3250). Gene *μ*, mutation rate for 1,067 bp of mtDNA *ND5* gene. YBP, years before present. Sources for shark rates: All sharks (Martin, Naylor & Palumbi, 1992; Dudgeon et al., 2012), *Raja* and *Aetobatus* (Dudgeon et al., 2012), *Mobula* (Poortvliet et al., 2015), and *Manta* (Kashiwagi et al., 2012).

	Substitution rate	Gene *μ*	Estimated split time (YBP)
All sharks	7.00E−10	7.47E−07	435074
*Raja*	4.21E−09	4.49E−06	72383
*Aetobatus*-slow	3.60E−09	3.84E−06	84635
*Aetobatus*-fast	6.35E−09	6.78E−06	47935
*Mobula*	8.82E−09	9.41E−06	34538
*Manta*	1.09E−08	1.16E−05	28017

## Results

### Environmental data

Manta rays were present in nearshore areas around Isla Holbox throughout the entire “manta season” (Fig. 1). We documented that in both years, August had the most numerous congregation of actively feeding mantas and, in fact, the majority of mantas found during the entire study were female (Fig. 2). Males were present in the highest numbers in May and July in both years; however, it is possible that males were in the area but were not present in the same large feeding aggregations as females.

**Figure 3:**
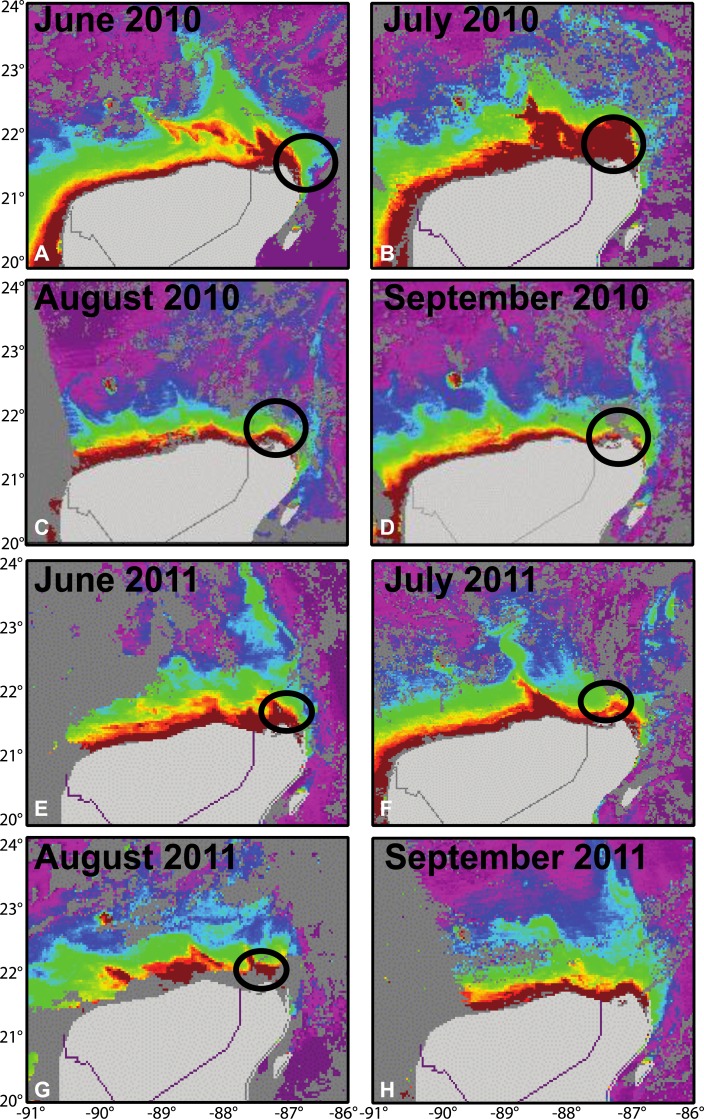
Monthly seasonal distribution of Chl-a during (A–D) May to September 2010 and (E–H) May to September 2011 based on data acquired from Modis-Aqua (NOAA). Red indicates the highest concentration of Chl-a recorded in the area. We have indicated areas where we have visually spotted manta rays feeding with a black oval. Note, we do not have visual identification of mantas for September 2011.

Yucatán Manta Rays move throughout established Marine Protected Areas, seemingly searching for the highest plankton concentrations (Fig. 3). This foraging behavior was confirmed by aerial surveys and sightings during “manta season” where, in the first two months, they were found close to the coast (Fig. 1). In July and August 2010, the mantas moved away from the coast due to an offshore shift in the rich nutrient waters; this was not observed in 2011 as mantas stayed northeast of Isla Contoy.

### Morphology

Previous visual identification guides for distinguishing between manta species rely on the relative size differences, dorsal and ventral coloration patterns, and the presence/absence of a reminiscent caudal spine (Marshall, Compagno & Bennett, 2009); however, this is unreliable as the morphometrics and coloration differences overlap between species. Yucatán Manta Rays are even more difficult to reliably identify by morphometrics as their size, color morphologies, and other morphometrics overlap with both *M. birostris* and *M. alfredi*. Further complicating matters, Yucatán Manta Rays and *M. birostris* habitats appear to overlap considerably in the Caribbean. Nonetheless we have created a visual guide (Table 3) to help identify between species in the field, but recommend that genetics be used in conjunction with visual identification when estimating populations, etc.

**Table 3:** *M. birostris*, *M. alfredi* and Yucatán Manta Rays basic morphology comparison. ✓, present; x, absent.

Morphology	Yucatán Manta Rays	*Manta birostris*	*Manta alfredi*
Embedded spine	✓	✓	x
Mouth color	White or Black	Black	White
Shoulder patches in supra-branchial region; triangular in shape	✓	✓	x
Shoulder patches in supra-branchial region that emanate posterior from the spiracle; curves similar to a whale tale	✓	x	✓
Dark spots between the five gill slits or pectoral fins	x	x	✓
Dorsal surface	Black or Brown	Black	Black

**Figure 4:**
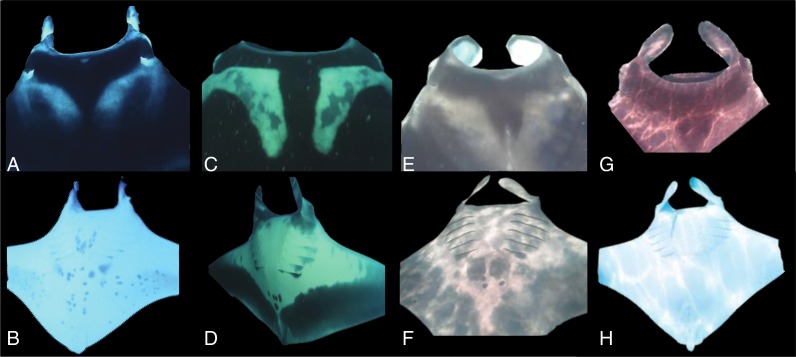
*Manta alfredi* (A) ventral and (B) dorsal surface; *Manta birostris* (C) ventral and (D) dorsal surface and Yucatán manta ray dorsal surface (E–G) and ventral surface (F–H).

*Manta birostris* (Figs. 4A and 4B) individuals have a black mouth, black dorsal surface with white shoulder patches in the supra-branchial region and in triangular shape. Ventral surface is cream to white with dark spots in the abdominal region and a calcified mass with an embedded spine (Marshall, Compagno & Bennett, 2009)

*Manta alfredi* (Figs. 4C and 4D) individuals have a white to light grey mouth, black dorsal surface, pale to white colored shoulder patches that emanate posteriorly from the spiracle before curving. The ventral surface is cream to white with a small black semi–circular spot located immediately posterior to the fifth gill. The calcified mass is not present (Marshall, Compagno & Bennett, 2009).

Yucatán Manta Rays have either a black (Figs. 4E and 4H) or white mouth, black or dark brown coloration on the dorsal surface, cream triangular shoulder patterns similar to *M. birostris* or white shoulder patches in “V” shape, similar to *M. alfredi*. The ventral surface is cream to white with no spots on the fifth gill slit and the ventral coloration varies from 60% black or grey coverage to 90% cream white and a calcified mass instead of a caudal spine.

**Figure 5:**
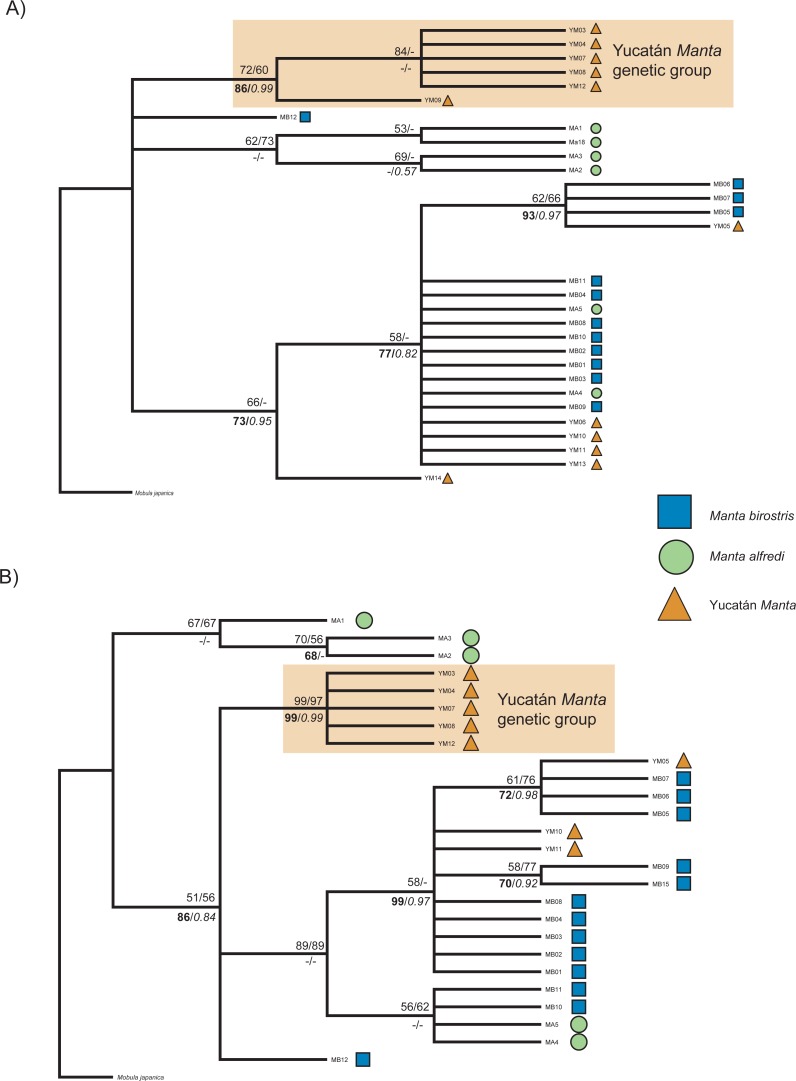
Neighbor-joining (NJ) phylogram with bootstrap values (NJ/Maximum Parsimony), above nodes, and bootstrap values/posterior probabilities (Maximum likelihood/Bayesian, respectively) below nodes. (A) 1,067 bp *ND5* dataset; (B) 1,136 bp *ND5* dataset.

**Figure 6:**
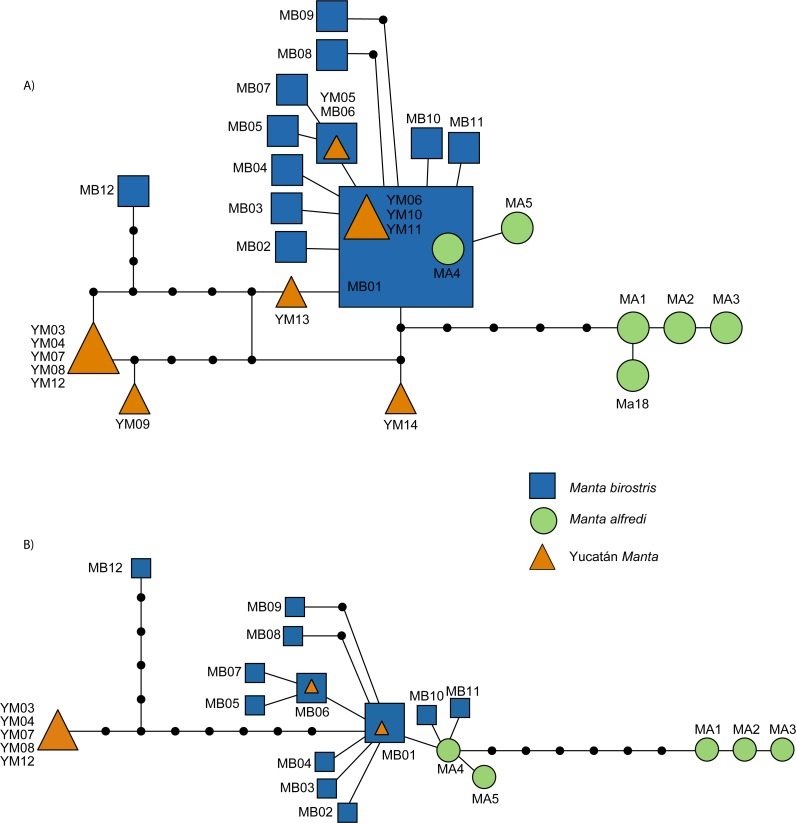
Statistical parsimony haplotype network of the mitochondrial *ND5* gene in *M. birostris* (Atlantic and Pacific), *M. alfredi* and Yucatán Manta Rays. (A) 1,067 bp dataset; (B) 1,136 bp dataset.

### Phylogenetic analysis

Among the Yucatán samples, four novel and exclusively Yucatán Manta Ray *ND5* haplotypes were recovered (YM03, YM09, YM13, YM14), and two shared Yucatán-*M. birostris- M. alfredi* haplotypes (YM05 same as MB06; YM06, YM10, YM11 same as MB01). Differences in the phylogenetic tree topologies among haplotypes were present for the various reconstruction methods. To best illustrate congruency across all methods, we showed a topology of the NJ-TrN+I tree generated by PAUP and map all bootstrap values and posterior probabilities onto the appropriate nodes (Fig. 5A). The highest support for a unique genetic clade was found among a subset of the Yucatán Manta Rays: YM09, YM03, YM04, YM07, YM08, and YM12 formed a well-resolved genetic group following Bayesian phylogenetic analyses (posterior probability = 0.99), and was further supported, at varying levels, with the other methods (Figs. 5A and 5B). The haplotype network recovered similar structure for relationships among *M. alfredi* and *M. birostris* as found in Kashiwagi et al. (2012), but further illustrates the differences among the four new Yucatán Manta Ray haplotypes and the other recognized *Manta* mtDNA groups (Figs. 6A and 6B). Of the new exclusive Yucatán haplotypes, YM03 and YM09 were more genetically divergent from the *Manta birostris* haplogroup (0.78% sequence divergence) compared to YM13 and YM14. Reliable DNA sequences were obtained from subset of Yucatán Manta Rays using the *RAG1* (YM03, YM04, YM05, YM07, YM08, YM09, YM12, YM13); however, all of these individuals showed the two nuclear SNPs consistent with *M. birostris* (A in position 73, C in position 507) as described by Kashiwagi et al. (2012).

**Figure 7:**
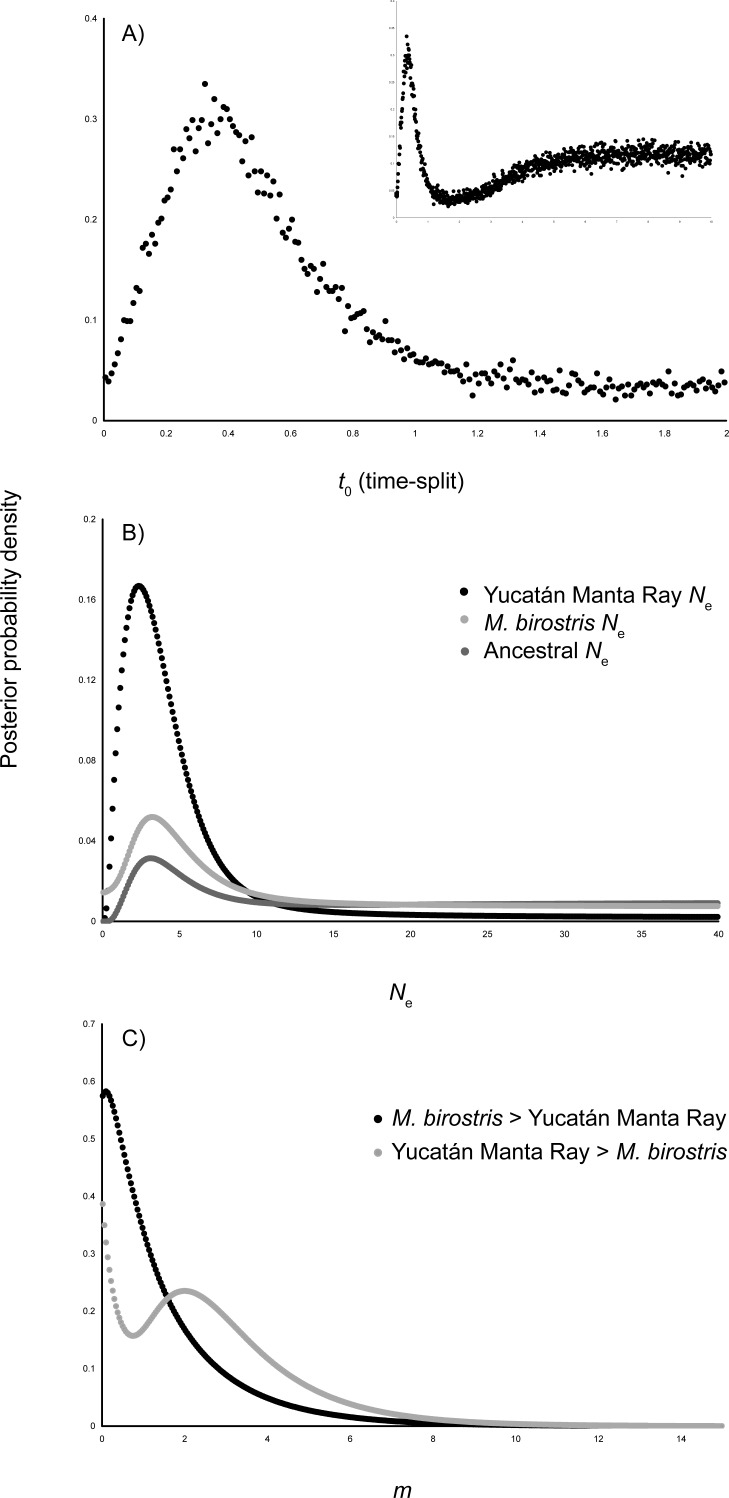
Posterior probabilities density curves for parameters estimated in IMa2. (A) Time-splitting scalar (t0) between Yucatán Manta Rays and *M. birostris*, (B) effective population size (*N*e), and (C) migration (*m*) between Yucatán Manta Ray and *M. birostris*.

All reported IMa2 parameter estimates are the Maximum-likelihood (peak of the marginal posterior probability distribution drawn from the L-mode run). The estimate for the time-splitting scalar (*t*_0_) was 0.3250 (Fig. 7A), which when scaled to the *Manta* specific mutation rate of Kashiwagi et al. (2012) and *Mobula* specific mutation rate of Poortvliet et al. (2015), yielded divergence times of 28,017 and 34,538 YBP among Yucatán Manta Rays and *M. birostris* (Table 3). Multiple comparisons of published elasmobranch mtDNA substitution rates were also evaluated, with *Aetobatus* and *Raja* rates producing fairly recent split times (<100,000 YBP), while the ‘All Sharks’ conservative rate of Martin, Naylor & Palumbi (1992) yielded a split at 435,074 (Table 3). Similar estimates of long-term effective population size (*N*_*e*_) were found for Yucatán Manta Rays, *M. birostris*, and the ancestral population (Fig. 7B). Evaluation of 25 demographic models in IMa2 L-mode yielded the highest support cases for the full model. Among the nested models, all supported asymmetrical migration from *M. birostris* into Yucatán Manta Rays (forward in time, Fig. 7C; and Table S1A) were not rejected using LLR tests (Supplemental Information). A model of equal coalescent effective population sizes between Yucatán Manta Rays and *M. birostris* (Model 6) was selected as the best fit following AIC (Table S1B). However ML estimates for migration in both directions resulted in marginal distributions that included zero (Fig. 7C). Runs performed not permitting migration yielded slightly earlier, but comparable split times (MLE *t*_0_ = 0.4050; 34,914 YBP and 43,039 YBP, following the *Manta* and *Mobula* rates, respectively). IM analysis of the 1,136 bp data set indicated a slightly earlier split time (*t*_0_ = 0.650, 56,034 YBP following the *Manta* specific rate).

## Discussion

### Genetic support

We have shown clear, genetic evidence supporting a third, distinctive manta genetic group using *ND5* (Figs. 5A and 5B; Figs. 6A and 6B). We note that while there are three (and possibly four) separate distinct genetic *Manta* haplotype groups, the group comprising *M. birostris* individuals (Yucatán samples presented herein, Kashiwagi et al., 2012; Walter et al., 2014) contained specimens that were visually identified as members from each morphospecies (e.g., *Manta birostris*, *Manta alfredi*, and the Yucatán Manta Ray). If we accept that *M. birostris* and *M. alfredi* are indeed two distinctive species (rather than ecomorphotypes or populations) with morphometric and genetic discordance, then this warrants recognition of the Yucatán Manta Ray as a third, distinctive genetic group—possibly an undescribed *Manta* species, or *M. birostris* subspecies.

The inclusion of individuals carrying *M. birostris* haplotypes but visually identified as either Yucatán Manta Rays (present study) or as *M. alfredi* from previous work (Kashiwagi et al., 2012; Walter et al., 2014) is inherently interesting because the opposite situation does not occur. To date there are no records of individuals visually identified as *M. birostris* having been shown to carry *M. alfredi* haplotypes, nor those of the Yucatán Manta Ray. We argue that this supports *M. birostris* as the likely ancestral manta to Yucatán Manta Rays, with the likelihood of ongoing or recent interspecific gene flow between these two *Manta* groups. Another possible explanation is that there is no selective pressure driving a change in phenotypic expression in the Yucatán Manta Ray or *M. alfredi*. However, a more plausible alternative is that morphologic and genetic differentiation distinguishing these groups is yet to be fully characterized (Walter et al., 2014), given such recent divergence among mantas (Kashiwagi et al., 2012, and this current study).

### Environmental support

We propose that if the divergent Yucatán Manta Ray genetic group represents a truly distinctive species, the mechanisms driving this separation from the other two manta ray species are likely similar to the proposed ecological barriers that led to the separation between *M. birostris* and *M. alfredi* (Kashiwagi et al., 2012). The speciation event for *M. birostris* and *M. alfredi* has been conservatively dated from 0.275 to 1 MYA during the Pleistocene (Kashiwagi et al., 2012). We hypothesize that Yucatán Manta Rays diverged in a much more recent time frame; conservative divergence time estimates based on our IMa analysis supports the notion a Yucatán Manta Ray-*M. birostris* split roughly between 28,000–56,000 YBP. These dates are consistent with the timing of the preceding last interglacial period, ∼125,000 YBP in the Pleistocene (Brunner, 1982; Ivanova, 2009) when sea levels were 4–6 m higher than the present (Dutton & Lambeck, 2012) with connectivity between the Gulf of Mexico and the Atlantic Ocean facilitating colonization of the Gulf by ancestral *M. birostris* individuals. The ensuing drop in global temperature leading up to the glacial maximum (18,000 YBP) resulted in a subsequent drop in global sea level, resulting in enlarged Floridian and Yucatán peninsula coastlines (Avise, 1992). These expanded coastlines could have restricted co-occurrence of *Manta* populations in the Gulf and Atlantic, thereby geographically isolating these populations. The general pattern of mtDNA variation between Yucatán Manta Rays and *M. birostris* is similar to those of other sister species, including several fish species, between the Gulf of Mexico and the Atlantic (Avise, 1992).

The Gulf Stream continues to play a definitive role in allopatric speciation of multiple marine animals including tropical fishes, echinoderms, and mollusks (Morin et al., 2010). For highly mobile species like *Manta* and *Mobula*, this softer geographic barrier is easily crossed; however, there may be other beneficial factors that contribute to reproductive and/or geographical isolation. These advantages may include, but are not limited to: (a) increased mating opportunities (Yano, Sato & Takahashi, 1999), (b) divergence in behavioral and/or sexual selection preferences (Edelaar, Siepielski & Clobert, 2008; Head et al., 2013; Poortvliet et al., 2015), (c) temporally consistent feeding opportunities (Hinojosa-Alvarez, 2009; Couturier et al., 2011), (d) favorable environmental conditions (Dewar et al., 2008; Clark, 2010; Graham et al., 2012), and (e) expanded coastal foraging opportunities (Notarbartolo di Sciara, 1988; Dewar et al., 2008; Marshall, Compagno & Bennett, 2009; Clark, 2010).

The highest concentration of Chl-a off the Yucatán Peninsula occurs in association with upwelling during May-September, as shown in the Aqua-Modis satellite images from NOAA (Fig. 3). This increased primary productivity would appear to provide favorable feeding opportunities for resident mantas. However, a substantial amount of evidence indicates that food is not a limiting factor for the Yucatán Manta Ray even in the winter months (Hinojosa-Alvarez, 2009; R. Friscione (www.pelagiclife.org/mantavalley). Environmental conditions remain favorable throughout the year with an average surface water temperature at 25 °C (www.nodc.noaa.gov), well within the 24–29 °C temperature preference measured for tagged mantas (Dewar et al., 2008; Graham et al., 2012). The recent tagging of nine Yucatán Manta Ray individuals (six tagged by Graham et al. (2012) and three by EM Paig-Tran, 2011, unpublished data; Microwave telemetry MK PAT) support the notion of a resident population that moves farther offshore during the winter months, yet still remains well within the boundaries of the Gulf of Mexico. The movements and habitat usage between *M. alfredi* and *M. birostris* differ in that the former has taken advantage of the new coastal foraging opportunities and remains more resident in select areas (Braun et al., 2015) while the latter is presumed a more oceanic and migratory species (Homma et al., 1999; Marshall, Compagno & Bennett, 2009; Deakos, Baker & Bejder, 2011). Nonetheless, recent work on *M. birostris* in Raja Ampat and Pacific México suggests more restricted movement (∼150 km radius; Stewart et al., 2016), indicating that characterization of *M. birostris* as highly migratory may be less than accurate for some populations. Given examples of residency in both species, it is not surprising that the Yucatán Manta Ray fills a similar nearshore niche around the Yucatán peninsula.

### Conclusions

Given that visual identification methods are not exclusively reliable in distinguishing between *Manta* species even among the most experienced field experts, we recommend morphological assessment coupled with genetic analyses, preferably at multiple loci, to avoid misidentification. We note that visual misidentifications are possible as *Manta* species maintain some level of overlap in their phenotypic descriptions and have been shown to change their coloration within minutes (Ari, 2014). However, individual manta rays can be reliably re-identified based on distinctive color patterns. Combining these data with an individual genetic identification may lead to further insights into their biology and population structures. More specifically, construction of a “genetic log” for resident individuals in areas where *Mantas* are highly monitored (e.g., Hawaii) may reveal levels of gene flow between *M. birostris* and the other *Manta* species and shed light on the reproductive viability of hybrid individuals.

Naming the Yucatán Manta Ray cannot yet be performed without careful inspection and genetic analyses of several museum specimens. Resurrection of the name *Cephaloptera giorna* Lesueur, 1824—updated to *Manta giorna*—would have precedence over the designation of *Manta americana* Bancroft, 1829; however it is unclear whether this specimen still exists. We note that Lesueur’s description of *Cephaloptera giorna* is consistent with the current description of Yucatán Manta Rays: presence of one to two serrated caudal spines in some specimens, disc widths ranging between 1.5 to 1.8 m, dark black to reddish dorsal coloration, a predominantly white ventral coloration with irregular spots, a tail longer than the body, and that they appear from July through September, probably correlating to the birthing season.

The holotype specimen of *Cephaloptera giorna* was collected from the mouth of the Delaware in Pennsylvania and a second specimen was described by Mitchill (1823) in New York as *Cephalopterus vampyrus*, which again matched similarly to the description of Yucatán Manta Rays including their characteristic brown dorsal coloration. It is unclear whether these mantas were indeed Yucatán Manta Rays as it is quite possible that these were simply *M. birostris* individuals that displayed overlapping external morphologies to Yucatán Manta Rays. Genetic confirmation of these individuals as Yucatán Manta Rays would greatly expand their distribution from the resident population proposed residing within the Caribbean. The designation of a genetically confirmed holotype specimen and a morphological and meristic redescription is necessary prior to formal taxonomic naming of this third species.

##  Supplemental Information

10.7717/peerj.2586/supp-1 Table S1A Joint peak locations and posterior probabilities for 25 models in L-mode IMa2. P-values obtained using 2LLR as Chi-Square statistic. Bold models were not rejected following LLR tests

10.7717/peerj.2586/supp-2 Table S1B AIC for models not-rejected following LLR tests from IMa2 L-mode run

10.7717/peerj.2586/supp-3 Supplemental Information 1 Manta identification

10.7717/peerj.2586/supp-4 Supplemental Information 2 Genbank accession numbers for ND5 Manta haplotypes

## Additional Information and Declarations

### Field Study Permissions

The following information was supplied relating to field study approvals (i.e., approving body and any reference numbers): Comision Nacional de Areas Naturales Protegidas (CONANP), Mexico and CONAPESCA No. DGOPA 00805.090210-0256.

### DNA Deposition

The following information was supplied regarding the deposition of DNA sequences: Genbank accession numbers
KR703227.1; FJ235625.1; KF574270.1; KR703232.1; KR703219.1; KR703228.1; KR703225.1; KR703226.1; KR703226.1; KR703229.1; KR703231.1; KR703233.1; KR703223.1; KR703222.1; KR703224.1; KR703220.1; KR703230.1; KR703218.1; KX291014; KX291015; KX291016; X350065; KX291017; KX291018; KX350064; KX291019; KX291020; KX291021; KX291022; KX291023.

### Data Availability

The following information was supplied regarding data availability: The raw data has been supplied as a Supplementary File.
