# Supplementary material for: A potential third Manta Ray species near the Yucatán Peninsula? Evidence for a recently diverged and novel genetic Manta group from the Gulf of Mexico
Source: PeerJ. 2016 Nov 1;4:e2586. doi: 10.7717/peerj.2586 (PMC5101608; doi:10.7717/peerj.2586)
Supplement: Table S1A — Joint peak locations and posterior probabilities for 25 models in L-mode IMa2. P-values obtained using 2LLR as Chi-Square statistic. Bold models were not rejected following LLR tests [file peerj-04-2586-s001.docx]

Supplemental Table 1a. Joint peak locations and posterior probabilities for 25 models in L-mode IMa2. P-values obtained using 2LLR as Chi-Square statistic. Bold models were not rejected following LLR tests.

|  |  |  |  |  |  |  |  |  |  |  |  |
| --- | --- | --- | --- | --- | --- | --- | --- | --- | --- | --- | --- |
| Model# | log(P) | #terms | df | 2LLR | ESS | q0 | q1 | q2 | m0>1 | m1>0 | P-value |
| 1 | -5.453 | 5 | - | - | 1.019 | 1.8635 | 2.1914 | 0.0046 | 1.9568 | 0 | - |
| 2 | -8.46 | 4 | 1 | 6.015 | 1.469 | 0.766 | 100 | 0.0285 | 5.3945 | [5.3945] | 0.014185 |
| 3 | -7.901 | 4 | 1* | 4.897 | 1.176 | 4.0707 | 100 | 0.0022 | [0.00000] | 2.4681 | 0.026903 |
| **4** | **-5.877** | **4** | **1** | **0.8491** | **1** | **0.4057** | **10.5091** | **0.4903** | **16.863** | **[0.00000]** | **0.356835** |
| 5 | -11.06 | 3 | 2* | 11.21 | 26.25 | 6.2042 | 100 | 3.1481 | [0.00000] | [0.00000] | 0.003679 |
| **6** | **-5.501** | **4** | **1** | **0.09635** | **1.02** | **2.0754** | **[2.0754]** | **0.0046** | **1.9586** | **0** | **0.756684** |
| **7** | **-7.585** | **3** | **2** | **4.265** | **1.015** | **4.019** | **[4.0190]** | **0.0016** | **0.1902** | **[0.1902]** | **0.118541** |
| 8 | -9.671 | 3 | 2* | 8.437 | 1.908 | 4.6975 | [4.6975] | 0.0022 | [0.00000] | 2.5439 | 0.014721 |
| **9** | **-5.501** | **3** | **2** | **0.09635** | **1.02** | **2.0754** | **[2.0754]** | **0.0046** | **1.9586** | **[0.00000]** | **0.953134** |
| 10 | -11.72 | 2 | 3* | 12.54 | 27.74 | 10.5722 | [10.5722] | 4.0762 | [0.00000] | [0.00000] | 0.005745 |
| **11** | **-5.904** | **4** | **1** | **0.9018** | **1.001** | **0.4299** | **10.5088** | **[0.4299]** | **16.8623** | **0** | **0.342246** |
| 12 | -9.005 | 3 | 2 | 7.105 | 435.5 | 1.3693 | 100 | [1.3693] | 3.2536 | [3.2536] | 0.028653 |
| 13 | -9.664 | 3 | 2* | 8.423 | 1123 | 4.7595 | 100 | [4.7595] | [0.00000] | 3.2461 | 0.014824 |
| **14** | **-5.904** | **3** | **2** | **0.9018** | **1.001** | **0.4299** | **10.5089** | **[0.4299]** | **16.8623** | **[0.00000]** | **0.636991** |
| 15 | -11.28 | 2 | 3* | 11.66 | 52.32 | 4.5043 | 100 | [4.5043] | [0.00000] | [0.00000] | 0.008643 |
| **16** | **-5.982** | **4** | **1** | **1.059** | **1.022** | **0.1203** | **9.3729** | **[9.3729]** | **26.2349** | **0.904** | **0.303443** |
| 17 | -9.697 | 3 | 2 | 8.488 | 1220 | 1.1478 | 100 | [100.0000] | 3.5898 | [3.5898] | 0.01435 |
| 18 | -10.09 | 3 | 2* | 9.27 | 1501 | 4.562 | 100 | [100.0000] | [0.00000] | 3.27 | 0.009706 |
| 19 | -8.151 | 3 | 2 | 5.396 | 363 | 3.6785 | 3.368 | [3.3680] | 0.8682 | [0.00000] | 0.06734 |
| 20 | -12.59 | 2 | 3* | 14.28 | 31.63 | 7.4644 | 6.006 | [6.0060] | [0.00000] | [0.00000] | 0.002548 |
| **21** | **-8.034** | **3** | **2** | **5.163** | **1.604** | **6.8809** | **[6.8809]** | **[6.8809]** | **33.0193** | **0.7446** | **0.07566** |
| 22 | -10.21 | 2 | 3 | 9.524 | 214.9 | 3.3904 | [3.3904] | [3.3904] | 0.422 | [0.4220] | 0.023077 |
| 23 | -10.29 | 2 | 3* | 9.683 | 494.4 | 5.1795 | [5.1795] | [5.1795] | [0.00000] | 3.6034 | 0.021462 |
| **24** | **-8.157** | **2** | **3** | **5.408** | **360.8** | **3.4971** | **[3.4971]** | **[3.4971]** | **0.8827** | **[0.00000]** | **0.144246** |
| 25 | -12.62 | 1 | 4* | 14.34 | 44.1 | 6.1552 | [6.1552] | [6.1552] | [0.00000] | [0.00000] | 0.006285 |
|  |  |  |  |  |  |  |  |  |  |  |  |
