# Supplementary material for: A potential third Manta Ray species near the Yucatán Peninsula? Evidence for a recently diverged and novel genetic Manta group from the Gulf of Mexico
Source: PeerJ. 2016 Nov 1;4:e2586. doi: 10.7717/peerj.2586 (PMC5101608; doi:10.7717/peerj.2586)
Supplement: Table S1B [file peerj-04-2586-s002.docx]

Supplemental Table 1b. AIC for models not-rejected following LLR tests from IMa2 L-mode run.

|  |  |  |
| --- | --- | --- |
| Model# | AIC | ΔAIC |
| 6 | 13.002 | 0 |
| 4 | 13.754 | 0.752 |
| 11 | 13.808 | 0.806 |
| 16 | 13.964 | 0.962 |
| 9 | 15.002 | 2 |
| 14 | 15.808 | 2.806 |
| 7 | 19.17 | 6.168 |
| 21 | 20.068 | 7.066 |
| 19 | 20.302 | 7.3 |
| 24 | 22.314 | 9.312 |
|  |  |  |
