# Supplementary figures and images for: A potential third Manta Ray species near the Yucatán Peninsula? Evidence for a recently diverged and novel genetic Manta group from the Gulf of Mexico
Source: PeerJ. 2016 Nov 1;4:e2586. doi: 10.7717/peerj.2586 (PMC5101608; doi:10.7717/peerj.2586)

Ventral surface for individual identification.


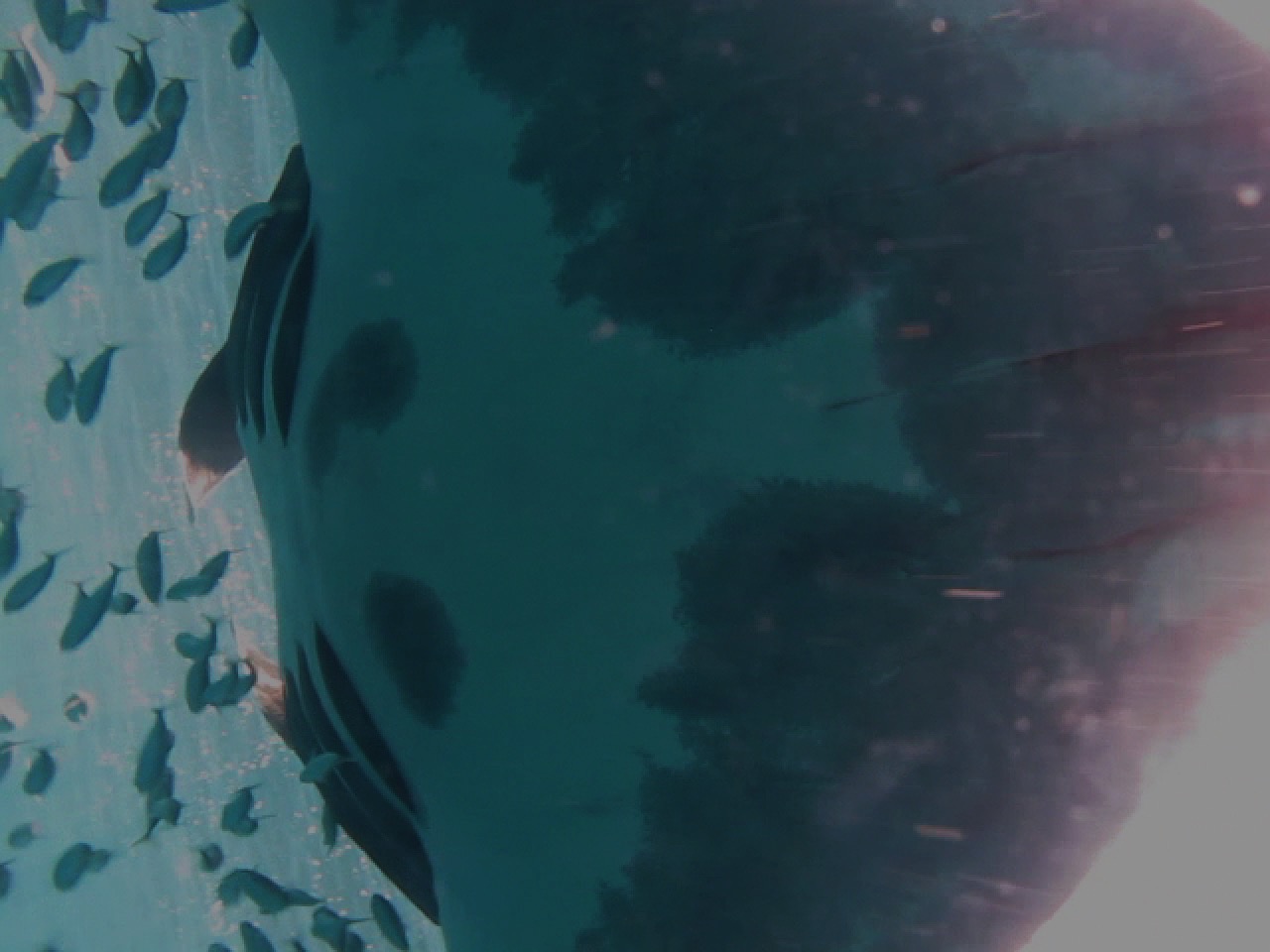

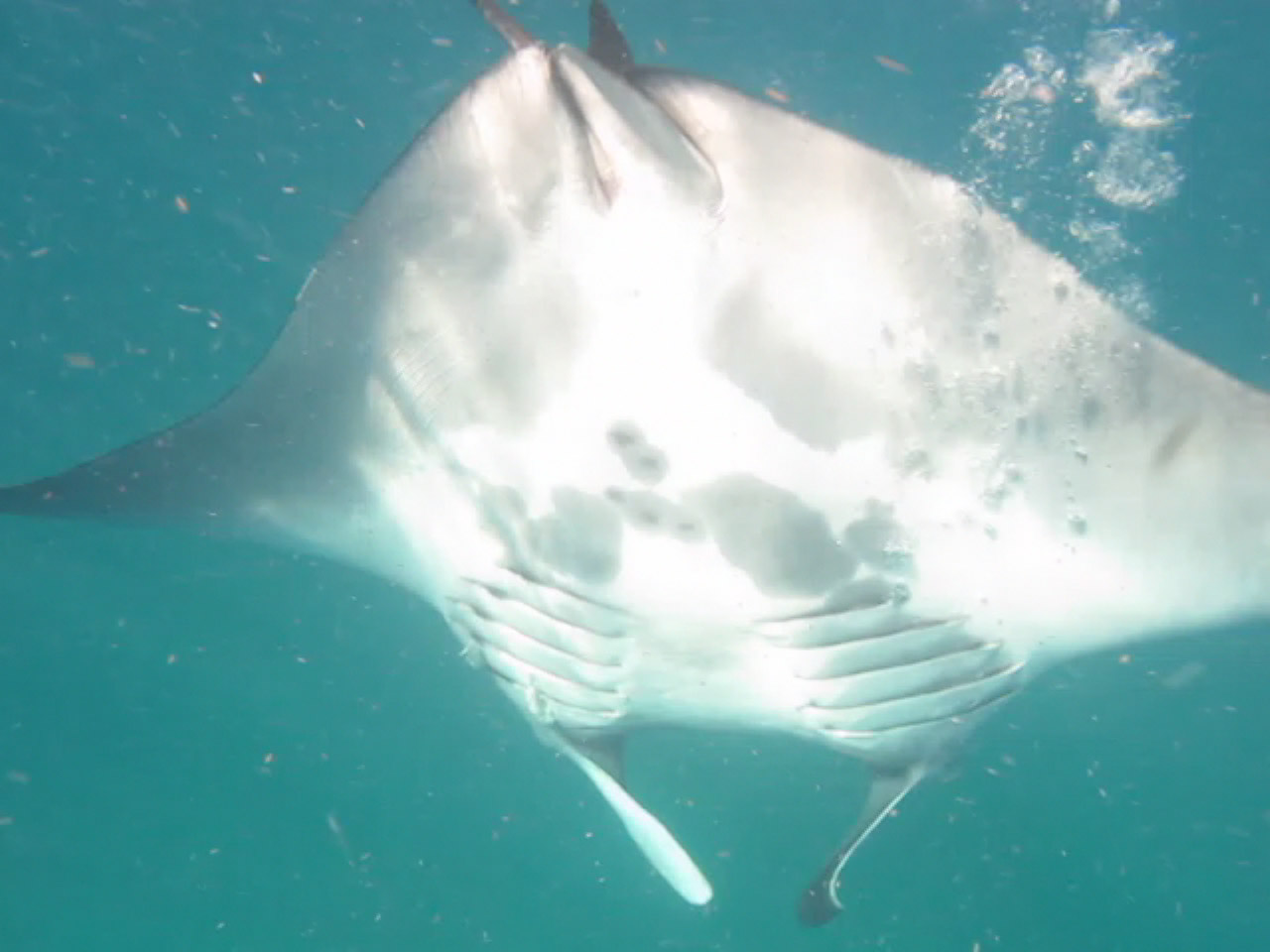

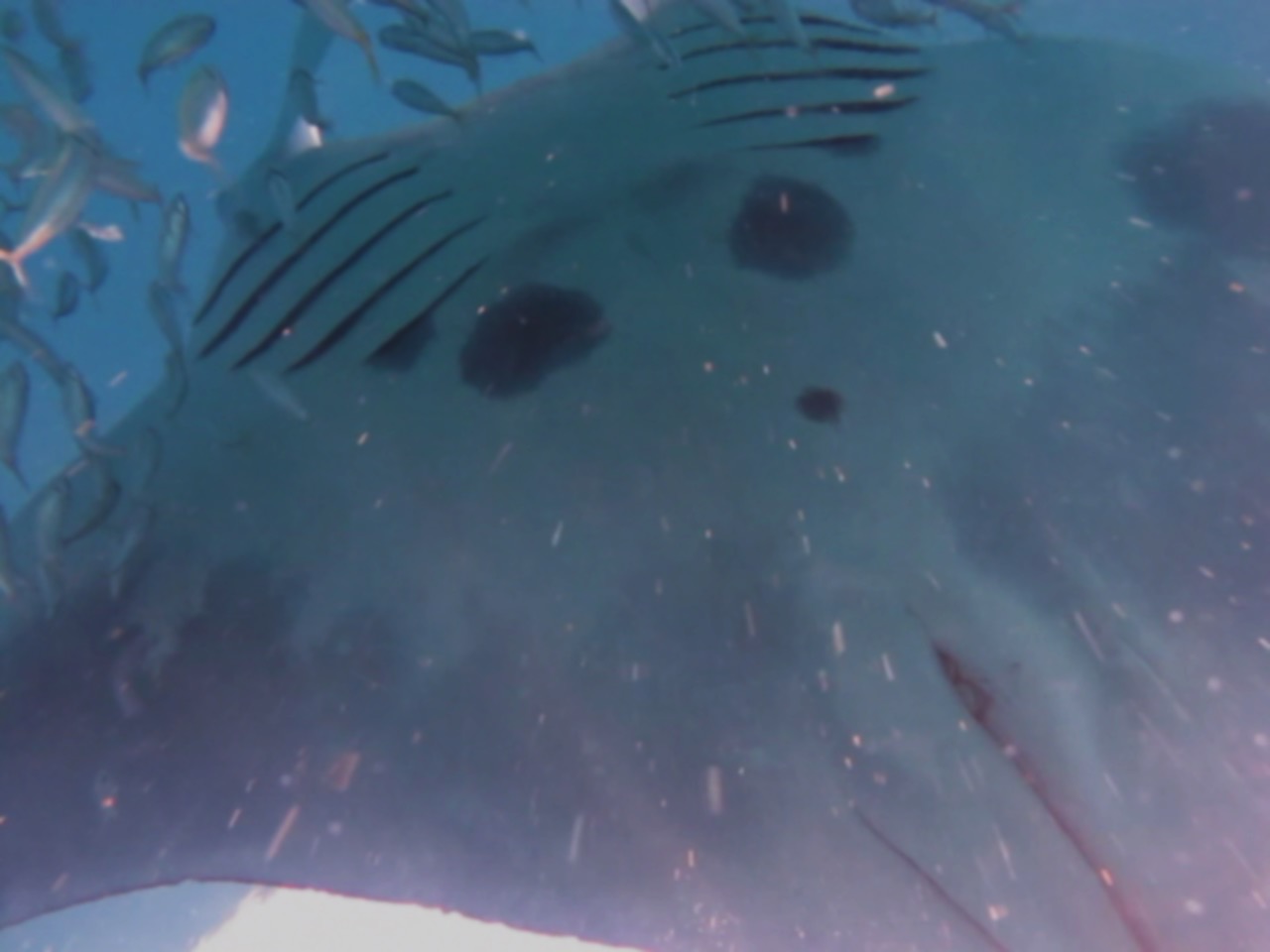


Hbx3 Hbx4 Hbx5


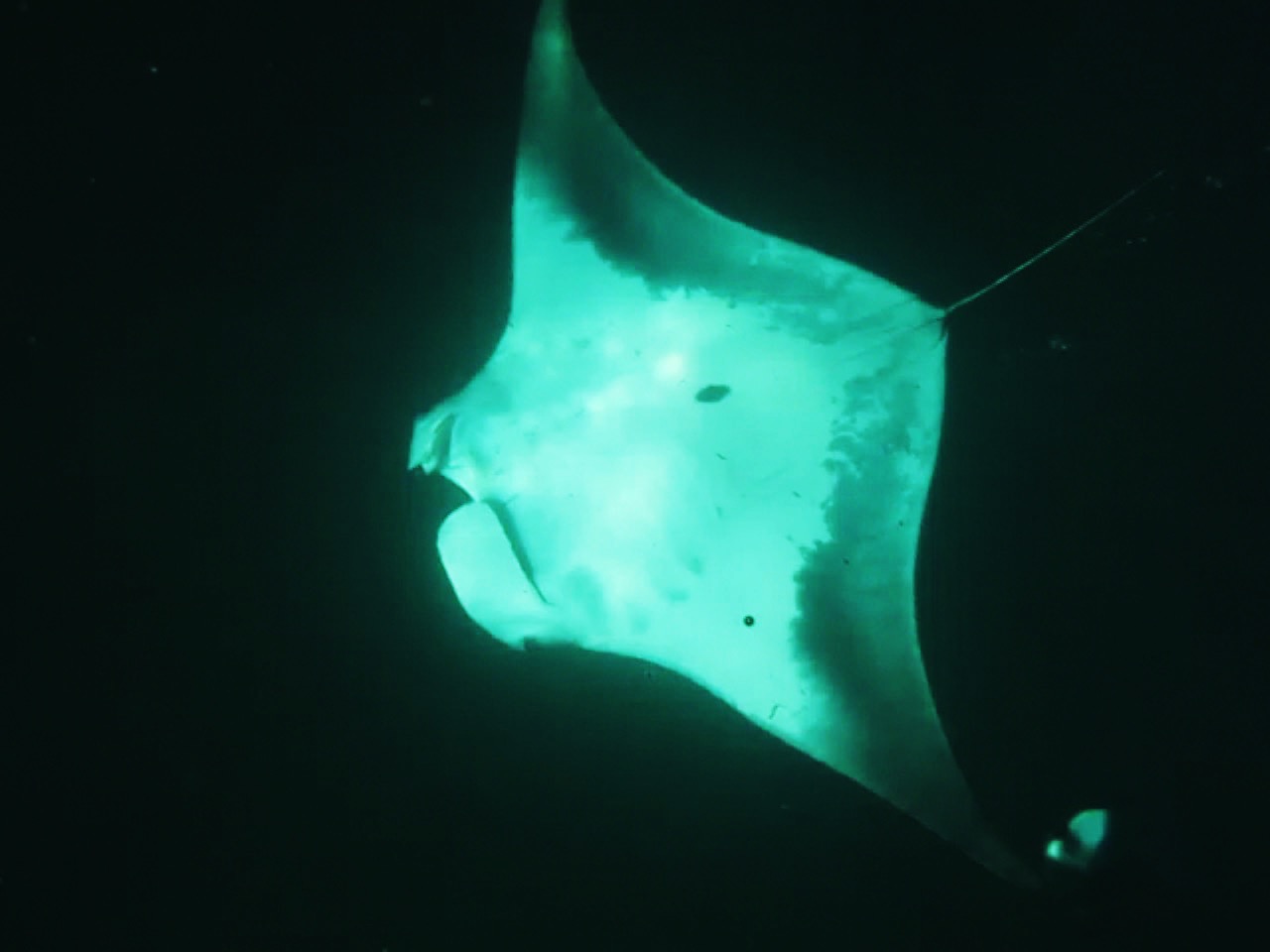

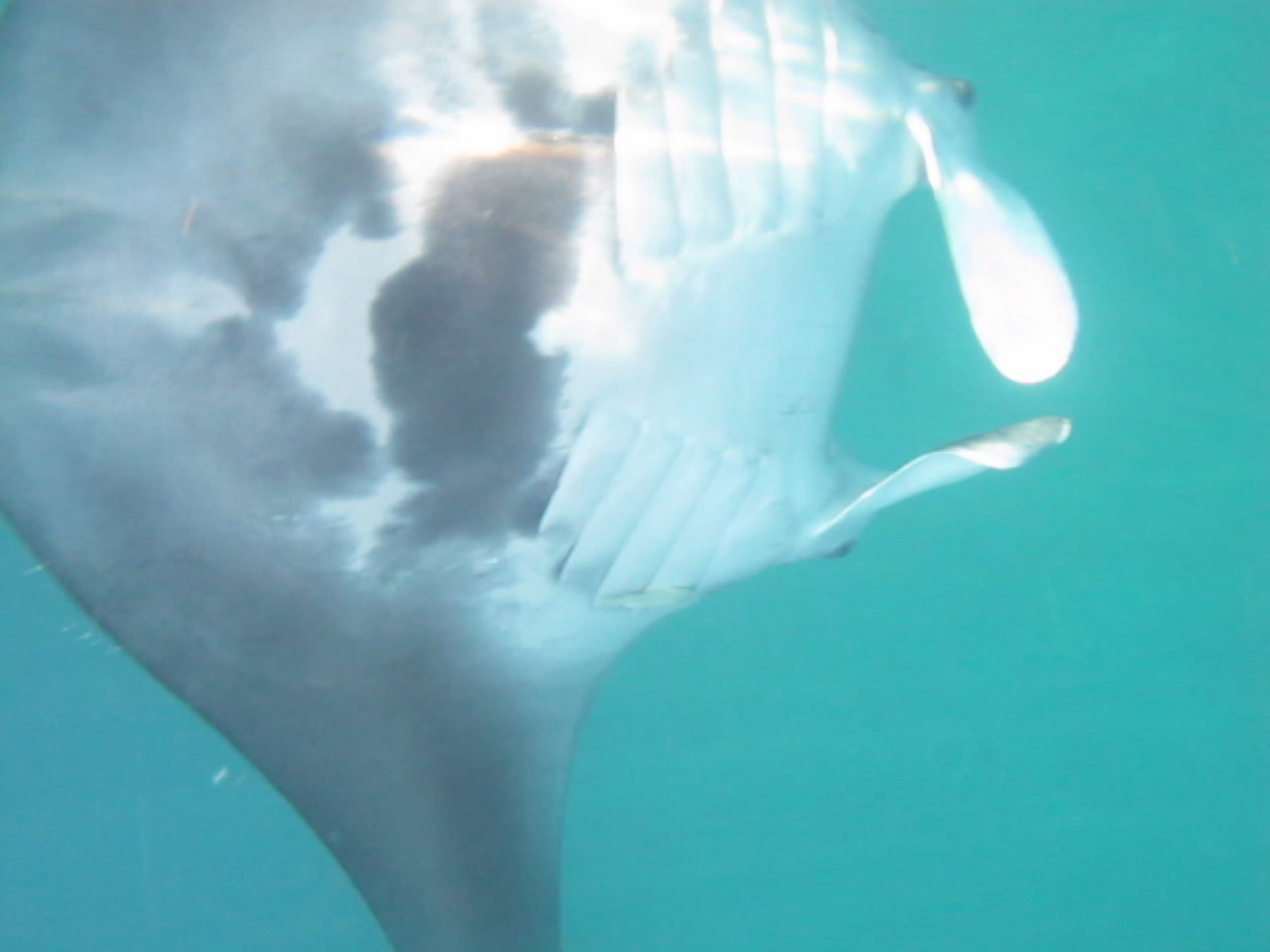

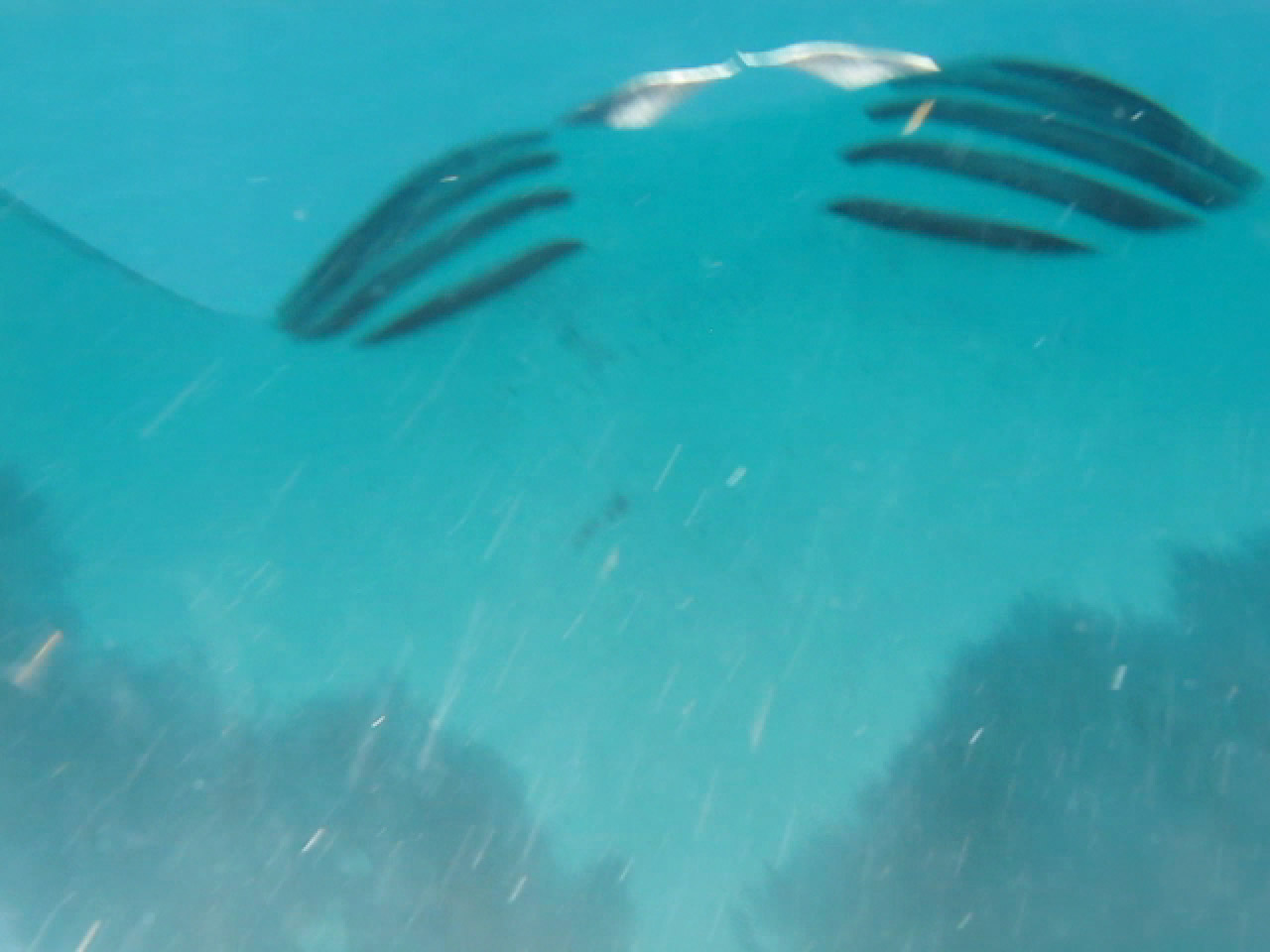


Hbx6 Hbx7 Hbx8


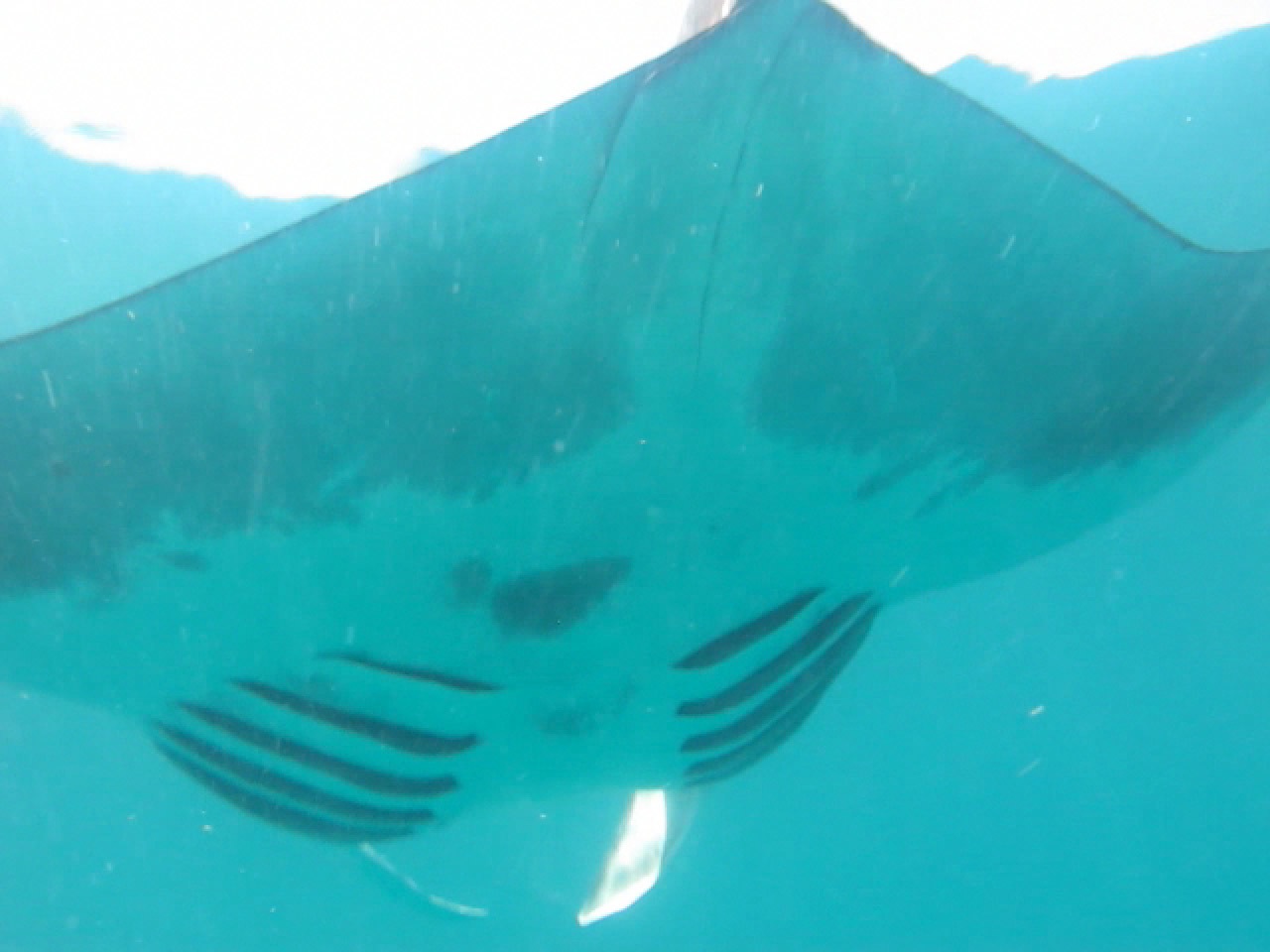

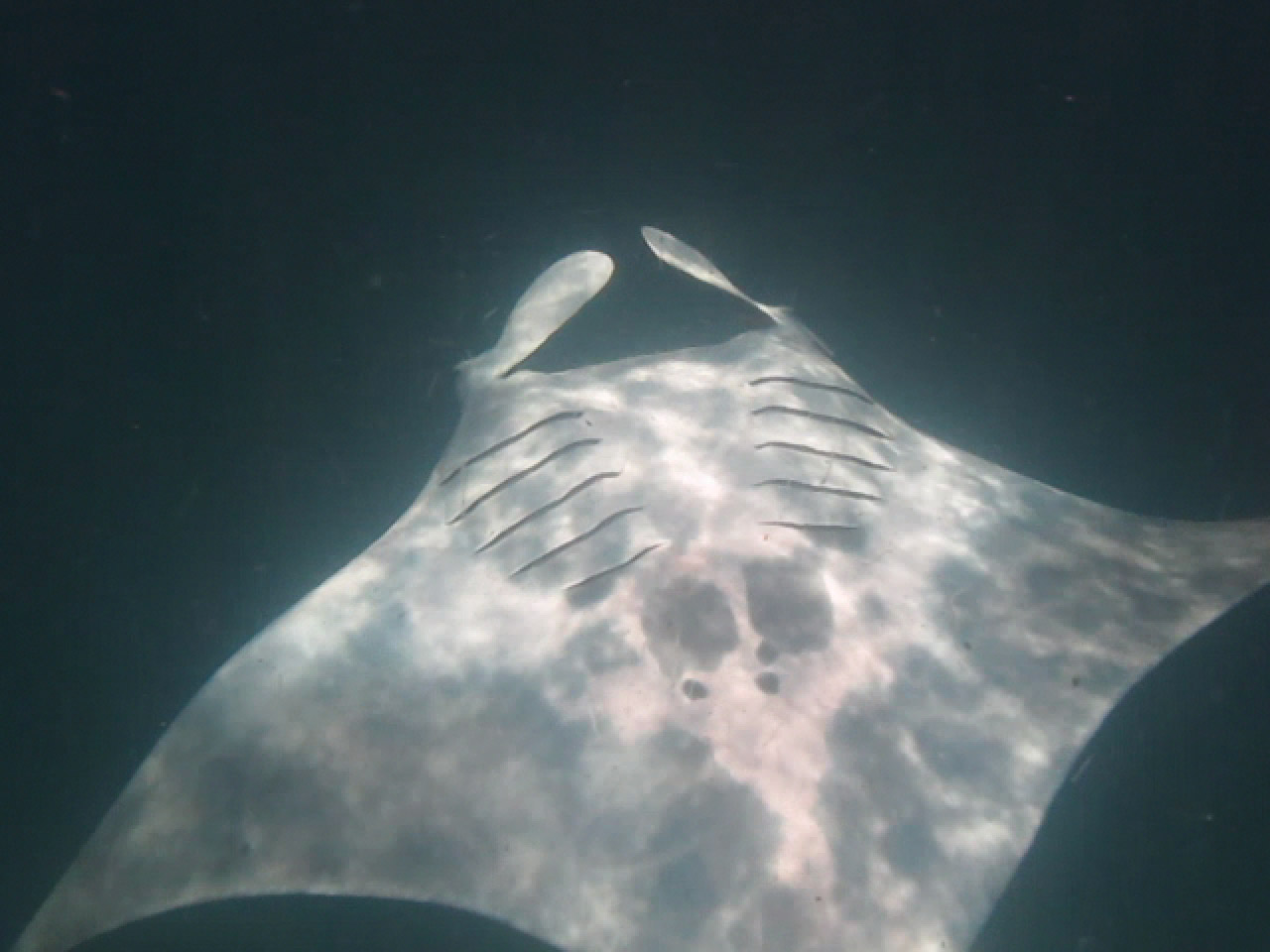

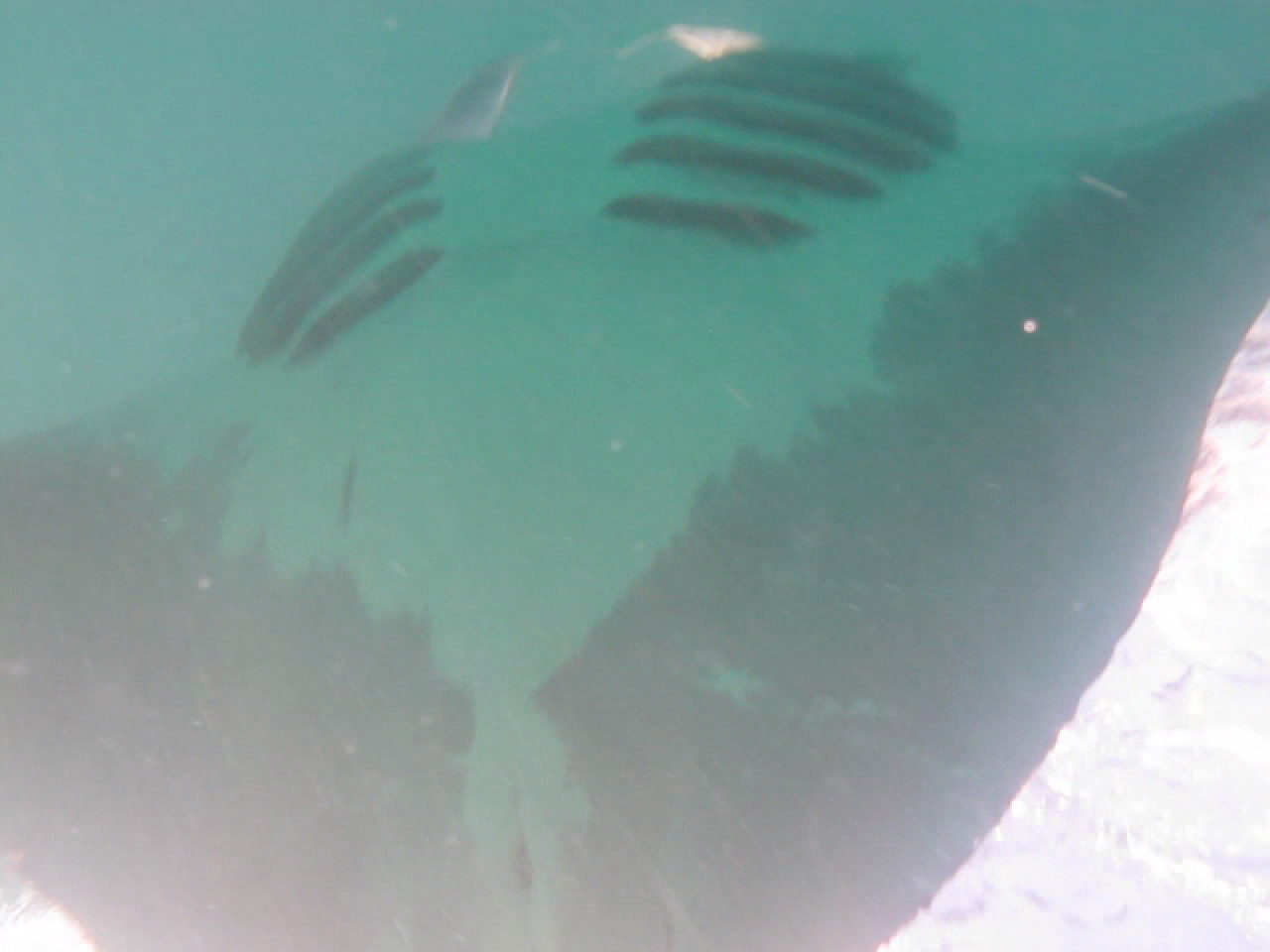


Hbx9 Hbx10 Hbx11


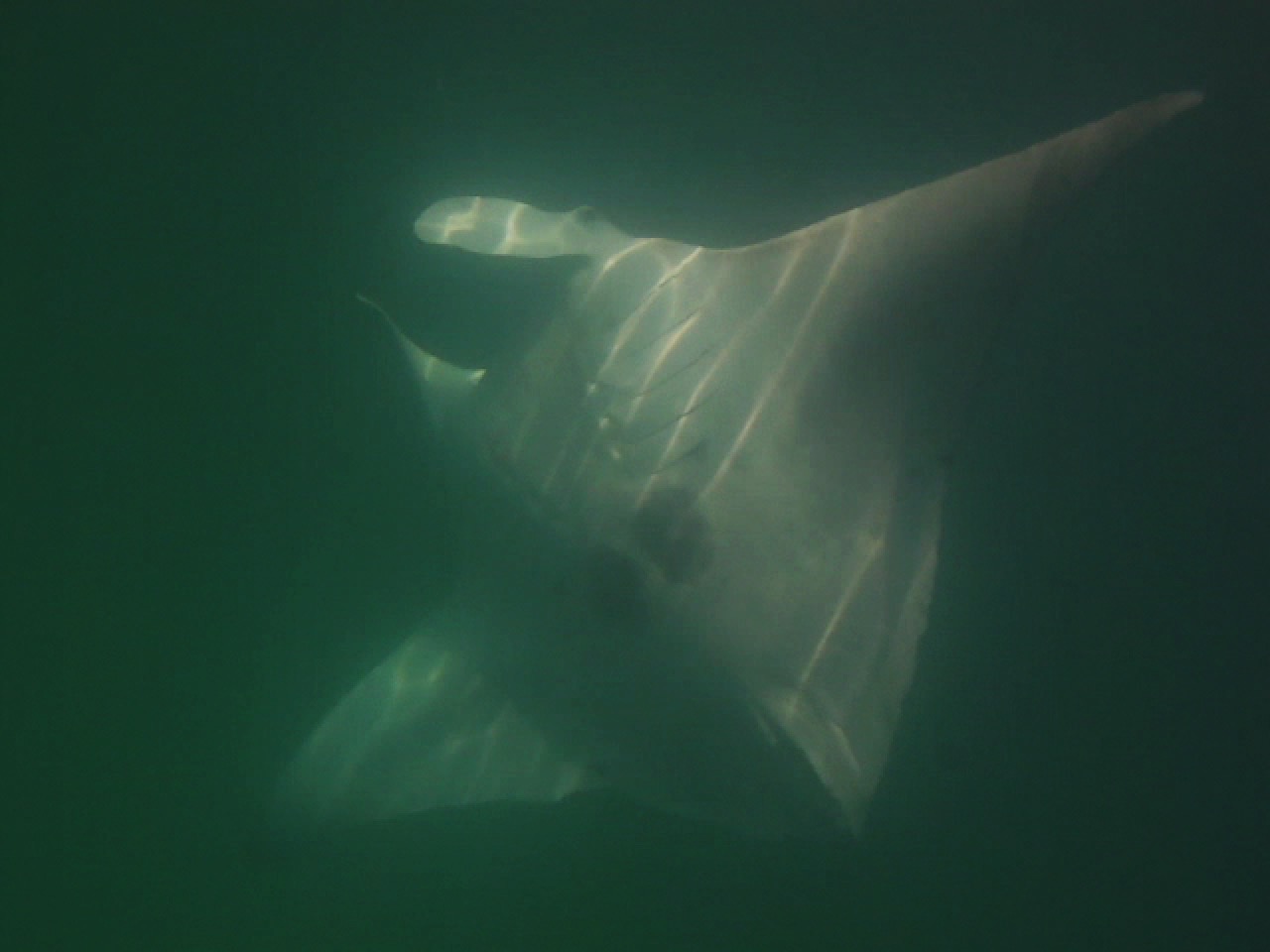

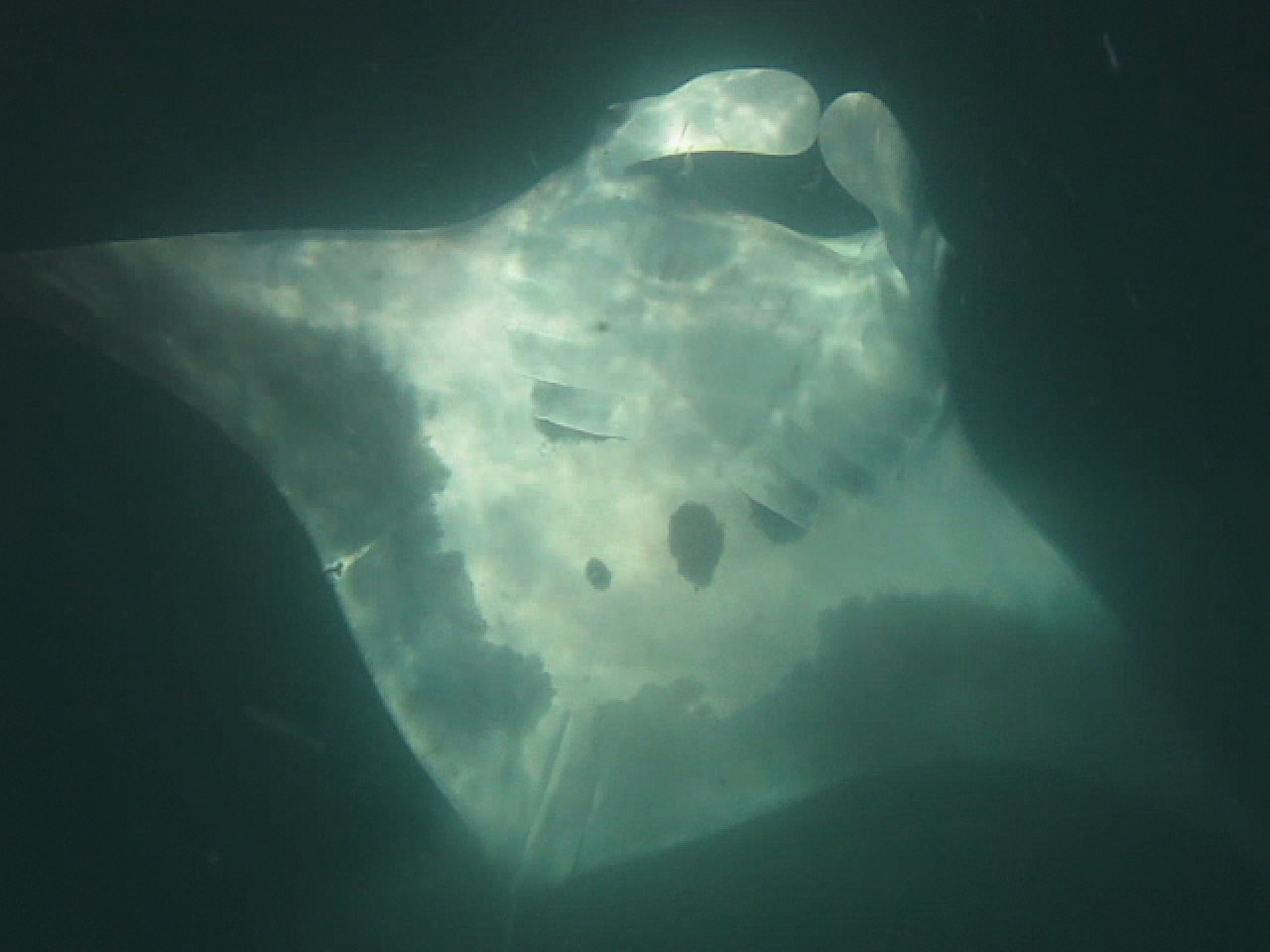


Hbx13 Hbx14

Supplement: Supplemental Information 1 [file peerj-04-2586-s003.docx]
