# Supplementary material for: A potential third Manta Ray species near the Yucatán Peninsula? Evidence for a recently diverged and novel genetic Manta group from the Gulf of Mexico
Source: PeerJ. 2016 Nov 1;4:e2586. doi: 10.7717/peerj.2586 (PMC5101608; doi:10.7717/peerj.2586)
Supplement: Supplemental Information 2 [file peerj-04-2586-s004.docx]

Supplementary_Genbank_1. Genbank accession numbers for ND5 Manta haplotypes

Sample Accesion number Sequence

MB06 KR703227.1 TTTTCCTAATCCTTTTATACCCTCTAATCTCATCCATACTTCCCACCAAAACATCACTTAACCACATTTGATCCTCCTCAGTCCATGTTAAAACAGCTGTAAAAACCTCTTTCTTTATTAGCCTTATCCCCTTATTTATCTTCTTAGACCAAGGTTTAGAATCCATCACAACCAACTGAAATTGATTCAACTTAGGGACCCTCGACATTAACATAAGTTTCAAATTTGACACCTATTCCATTATCTTCACTCCCGTAGCCCTATATGTAACCTGATCTATCCTAGAATTTGCCTTATGATATATGCACTCAGACCCCAACCTAAGTAAATTCTTTAAATACCTTTTATTGTTCTTAATCACAATACTAATCCTCATTACAGCTAATAACCTATTCCAACTATTCATCGGTTGAGAGGGTGTAGGAATTATATCATTCCTCCTCATTGGCTGATGATTCAGCCGAACCGATGCAAACACAGCTGCTCTCCAAGCCGTTATCTATAACCGTATCGGAGATATCGGTTTAATCATAGCCATAGCATGACTAGCCATAAACCTAAACTCATGGGAGATACAACAACTCTTCTTTCTCTCCAAAAACACAAATCTAACCCTTCCGCTTCTAGGGTTAGTTCTAGCAGCAGCTGGAAAATCAGCCCAATTCGGCCTACACCCATGACTTCCAGCCGCCATAGAGGGTCCTACGCCAGTCTCTGCCCTACTTCACTCAAGCACAATAGTAGTTGCAGGTGTCTTCCTACTTATCCGCCTCCACCCTTTAATTCAAGACAACCATGTAATTCTATCAACCTGCCTATGTCTAGGGGCACTTACAACCCTCTTCACAGCTACCTGTGCACTCACCCAAAACGACATCAAAAAAATTATCGCTTTCTCTACATCTAGCCAACTAGGTCTTATAATAGTCACTATTGGTCTCAACCAACCACAGTTGGCCTTCCTCCACATCTGCACACACGCCTTCTTCAAAGCAATGCTCTTCCTATGCTCTGGCTCCATCATCCACAGCCTAAATGATGAACAAGATATCCGAAAAATAGGGGGC

MAH1 FJ235625.1 TTTTCCTAATCCTTTTATATCCTCTAATCTCATCCATACTTCCCACCAAAACATCACTTAACCACATTTGATCCTCCTCAGTCCATGTCAAAACAGCTGTAAAAACCTCTTTCTTTATTAGCCTTATTCCCTTATTTATCTTCTTAGACCAAGGTTTAGAATCCATCACAACCAACTGAAATTGATTCAACTTAGGGACCCTCGACATTAACATAAGTTTCAAATTTGACACCTATTCCATTATCTTCACTCCCGTAGCCCTATATGTAACCTGATCTATCCTAGAATTTGCCTTATGATATATGCACTCAGACCCCAACCTAAGTAAATTCTTTAAATACCTTTTATTGTTCTTAATCACAATACTAATCCTCATTACAGCTAATAACCTATTCCAACTATTCATCGGTTGAGAAGGTGTAGGAATTATATCATTCCTCCTCATTGGCTGATGATTCAGCCGAACCGATGCAAACACAGCTGCTCTCCAAGCCGTTATCTATAACCGTATCGGAGATATCGGTTTAATCATAGCCATAGCATGACTAGCCATAAACCTAAACTCATGGGAGATACAACAACTCTTCTTTCTCTCCAAAAACACAAATCTAACCCTTCCACTTCTAGGGTTAGTTCTAGCAGCAGCTGGAAAATCAGCCCAATTCGGCCTACACCCATGACTTCCAGCCGCCATAGAGGGTCCTACGCCAGTCTCTGCCCTACTTCACTCAAGCACAATAGTAGTTGCAGGTGTCTTCCTACTTATCCGCCTCCACCCTTTAATTCAAGACAACCATGTAATTCTATCAACCTGCCTATGTCTAGGGGCACTTACAACCCTCTTCACAGCTACCTGTGCACTCACCCAGAACGATATCAAAAAAATTATCGCTTTCTCTACATCTAGCCAACTAGGTCTTATAATAGTCACTATTGGTCTCAACCAACCACAGTTGGCCTTCCTCCACATCTGCACACACGCCTTCTTCAAAGCAATGCTCTTCCTATGCTCTGGCTCCATCATCCACAGCCTAAATGATGAACAAGATATCCGAAAAATAGGGGGC

Ma18 KF574270.1 TTTTCCTAATCCTTTTATATCCCCTAATCTCATCCATACTTCCCACCAAAACATCACTTAACCACATTTGATCCTCCTCAGTCCATGTCAAAACAGCTGTAAAAACCTCTTTCTTTATTAGCCTTATTCCCTTATTTATCTTCTTAGACCAAGGTTTAGAATCCATCACAACCAACTGAAATTGATTCAACTTAGGGACCCTCGACATTAACATAAGTTTCAAATTTGACACCTATTCCATTATCTTCACTCCCGTAGCCCTATATGTAACCTGATCTATCCTAGAATTTGCCTTATGATATATGCACTCAGACCCCAACCTAAGTAAATTCTTTAAATACCTTTTATTGTTCTTAATCACAATACTAATCCTCATTACAGCTAATAACCTATTCCAACTATTCATCGGTTGAGAAGGTGTAGGAATTATATCATTCCTCCTCATTGGCTGATGATTCAGCCGAACCGATGCAAACACAGCTGCTCTCCAAGCCGTTATCTATAACCGTATCGGAGATATCGGTTTAATCATAGCCATAGCATGACTAGCCATAAACCTAAACTCATGGGAGATACAACAACTCTTCTTTCTCTCCAAAAACACAAATCTAACCCTTCCACTTCTAGGGTTAGTTCTAGCAGCAGCTGGAAAATCAGCCCAATTCGGCCTACACCCATGACTTCCAGCCGCCATAGAGGGTCCTACGCCAGTCTCTGCCCTACTTCACTCAAGCACAATAGTAGTTGCAGGTGTCTTCCTACTTATCCGCCTCCACCCTTTAATTCAAGACAACCATGTAATTCTATCAACCTGCCTATGTCTAGGGGCACTTACAACCCTCTTCACAGCTACCTGTGCACTCACCCAGAACGATATCAAAAAAATTATCGCTTTCTCTACATCTAGCCAACTAGGTCTTATAATAGTCACTATTGGTCTCAACCAACCACAGTTGGCCTTCCTCCACATCTGCACACACGCCTTCTTCAAAGCAATGCTCTTCCTATGCTCTGGCTCCATCATCCACAGCCTAAATGATGAACAAGATATCCGAAAAATAGGGGGC

MB11 KR703232.1 TTTTCCTAATCCTTTTATACCCTCTAATCTCATCCATACTTCCCACCAAAACATCACTTAACCACATTTGATCCTCCTCAGTCCATGTTAAAACAGCTGTAAAAACCTCTTTCTTTATTAGCCTTATCCCCTTATTTATCTTCTTAGACCAAGGTTTAGAATCCATCACAACCAACTGAAATTGATTCAACTTAGGGACCCTCGACATTAACATAAGTTTCAAATTTGACACCTATTCCATTATCTTCACTCCCGTAGCCCTATATGTAACCTGATCTATCCTAGAATTTGCCTTATGATATATGCACTCAGACCCCAACCTAAGTAAATTCTTTAAATACCTTTTATTGTTCTTAATCACAATACTAATCCTCATTACAGCTAATAACCTATTCCAACTATTCATCGGTTGAGAAGGTGTAGGAATTATATCATTCCTCCTCATTGGCTGATGATTCAGCCGAACCGATGCAAACACAGCTGCTCTCCAAGCCGTTATCTATAACCGTATCGGAGATATCGGTTTAATCATAGCCATAGCATGACTAGCCATAAACCTAAACTCATGGGAGATGCAACAACTCTTCTTTCTCTCCAAAAACACAAATCTAACCCTTCCGCTTCTAGGGTTAGTTCTAGCAGCAGCTGGAAAATCAGCCCAATTCGGCCTACACCCATGACTTCCAGCCGCCATAGAGGGTCCTACGCCAGTCTCTGCCCTACTTCACTCAAGCACAATAGTAGTTGCAGGTGTCTTCCTACTTATCCGCCTCCACCCTTTAATTCAAGACAACCATGTAATTCTATCAACCTGCCTATGTCTAGGGGCACTTACAACCCTCTTCACAGCTACCTGTGCACTCACCCAAAACGACATCAAAAAAATTATCGCTTTCTCTACATCTAGCCAACTAGGTCTTATAATAGTCACTATTGGTCTCAACCAACCACAGTTGGCCTTCCTCCACATCTGCACACACGCCTTCTTCAAAGCAATGCTCTTCCTATGCTCTGGCTCCATCATCCACAGCCTAAATGATGAACAAGATATCCGAAAAATAGGGGGC

MAH3 KR703219.1 TTTTCCTAATCCTTTTATATCCTCTAATCTCATCCATACTTCCCACCAAAACATCACTTAACCACATTTGATCCTCCTCAGTCCATGTCAAAACAGCTGTAAAAACCTCTTTCTTTATTAGCCTTATTCCCTTATTTATCTTCTTAGACCAAGGTTTAGAATCCATCACAACCAACTGAAATTGATTCAACTTAGGGACCCTCGACATTAACATAAGTTTCAAATTTGACACCTATTCCATTATCTTCACTCCCGTAGCCCTATATGTAACCTGATCTATCCTAGAATTTGCCTTATGATATATGCACTCAGACCCCAACCTAAGTAAATTCTTTAAATACCTTTTATTGTTCTTAATCACAATACTAATCCTCATTACAGCTAATAACCTACTCCAACTATTCATCGGTTGAGAAGGTGTAGGAATTATATCATTCCTCCTCATTGGCTGATGATTCAGCCGAACCGATGCAAACACAGCTGCTCTCCAAGCCGTTATCTATAACCGTATCGGAGATATCGGTTTAATCATAGCCATAGCATGACTAGCCATAAACCTAAACTCATGGGAGATACAACAACTCTTCTTTCTCTCCAAAAACACAAATCTAACCCTTCCACTTCTAGGGTTAGTTCTAGCAGCAGCTGGAAAATCAGCCCAATTCGGCCTACACCCATGACTTCCAGCCGCCATAGAGGGTCCTACGCCAGTCTCTGCCCTACTTCACTCAAGCACAATAGTAGTTGCAGGTGTCTTCCTACTTATCCGCCTCCACCCTTTAATTCAAGACAACCATGTAATTCTATCAACCTGCCTATGTCTAGGGGCACTTACAACCCTCTTCACAGCTACCTGTGCACTCACCCAGAACGATATCAAAAAAATTATCGCTTTCTCTACATCTAGCCAACTAGGTCTTATAATAGTCACTATTGGTCTCAACCAACCACAATTGGCCTTCCTCCACATCTGCACACACGCCTTCTTCAAAGCAATGCTCTTCCTATGCTCTGGCTCCATCATCCACAGCCTAAATGATGAACAAGATATCCGAAAAATAGGGGGC

MB07 KR703228.1 TTTTCCTAATCCTTTTATACCCTCTAATCTCATCCATACTTCCCACCAAAACATCACTTAACCACATTTGATCCTCCTCAGTCCATGTTAAAACAGCTGTAAAAACCTCTTTCTTTATTAGCCTTATCCCCTTATTTATCTTCTTAGACCAAGGTTTAGAATCCATCACAACCAACTGAAATTGATTCAACTTAGGGACCCTCGACATTAACATAAGTTTCAAATTTGACACCTATTCCATTATCTTCACTCCCGTAGCCCTATATGTAACCTGATCTATCCTAGAATTTGCCTTATGATATATGCACTCAGACCCCAACCTAAGTAAATTCTTTAAATACCTTTTATTGTTCTTAATCACAATACTAATCCTCATTACAGCTAATAACCTATTCCAACTATTCATCGGTTGAGAGGGTGTAGGAATTATATCATTCCTCCTCATTGGCTGATGATTCAGCCGAACCGATGCAAACACAGCTGCTCTCCAAGCCGTTATCTATAACCGTATCGGAGATATCGGTTTAATCATAGCCATAGCATGACTAGCCATAAACCTAAACTCATGGGAGATACAACAACTCTTCTTTCTCTCCAAAAACACAAATCTAACCCTTCCGCTTCTAGGGTTAGTTCTAGCAGCAGCTGGAAAATCAGCCCAATTCGGCCTACACCCATGACTTCCAGCCGCCATAGAGGGTCCTACGCCAGTCTCTGCCCTACTTCACTCAAGCACAATAGTAGTTGCAGGTGTCTTCCTACTTATCCGCCTCCACCCTTTAATTCAAGACAACCATGTAATTCTATCAACCTGCCTATGTCTGGGGGCACTTACAACCCTCTTCACAGCTACCTGTGCACTCACCCAAAACGACATCAAAAAAATTATCGCTTTCTCTACATCTAGCCAACTAGGTCTTATAATAGTCACTATTGGTCTCAACCAACCACAGTTGGCCTTCCTCCACATCTGCACACACGCCTTCTTCAAAGCAATGCTCTTCCTATGCTCTGGCTCCATCATCCACAGCCTAAATGATGAACAAGATATCCGAAAAATAGGGGGC

MB04 KR703225.1 TTTTCCTAATCCTTTTATACCCTCTAATCTCATCCATACTTCCCACCAAAACATCACTTAACCACATTTGATCCTCCTCAGTCCATGTTAAAACAGCTGTAAAAACCTCTTTCTTTATTAGCCTTATCCCCTTATTTATCTTCTTAGATCAAGGTTTAGAATCCATCACAACCAACTGAAATTGATTCAACTTAGGGACCCTCGACATTAACATAAGTTTCAAATTTGACACCTATTCCATTATCTTCACTCCCGTAGCCCTATATGTAACCTGATCTATCCTAGAATTTGCCTTATGATATATGCACTCAGACCCCAACCTAAGTAAATTCTTTAAATACCTTTTATTGTTCTTAATCACAATACTAATCCTCATTACAGCTAATAACCTATTCCAACTATTCATCGGTTGAGAAGGTGTAGGAATTATATCATTCCTCCTCATTGGCTGATGATTCAGCCGAACCGATGCAAACACAGCTGCTCTCCAAGCCGTTATCTATAACCGTATCGGAGATATCGGTTTAATCATAGCCATAGCATGACTAGCCATAAACCTAAACTCATGGGAGATACAACAACTCTTCTTTCTCTCCAAAAACACAAATCTAACCCTTCCGCTTCTAGGGTTAGTTCTAGCAGCAGCTGGAAAATCAGCCCAATTCGGCCTACACCCATGACTTCCAGCCGCCATAGAGGGTCCTACGCCAGTCTCTGCCCTACTTCACTCAAGCACAATAGTAGTTGCAGGTGTCTTCCTACTTATCCGCCTCCACCCTTTAATTCAAGACAACCATGTAATTCTATCAACCTGCCTATGTCTAGGGGCACTTACAACCCTCTTCACAGCTACCTGTGCACTCACCCAAAACGACATCAAAAAAATTATCGCTTTCTCTACATCTAGCCAACTAGGTCTTATAATAGTCACTATTGGTCTCAACCAACCACAGTTGGCCTTCCTCCACATCTGCACACACGCCTTCTTCAAAGCAATGCTCTTCCTATGCTCTGGCTCCATCATCCACAGCCTAAATGATGAACAAGATATCCGAAAAATAGGGGGC

MA05 KR703226.1 TTTTCCTAATCCTTTTATACCCTCTAATCTCATCCATACTTCCCACCAAAACATCACTTAACCACATTTGATCCTCCTCAGTCCATGTTAAAACAGCTGTAAAAACCTCTTTCTTTATTAGCCTTATCCCCTTATTTATCTTCTTAGACCAAGGTTTAGAATCCATCACAACCAACTGAAATTGATTCAACTTAGGGACCCTCGACATTAACATAAGTTTCAAATTTGACACCTATTCCATTATCTTCACTCCCGTAGCCCTATATGTAACCTGATCTATCCTAGAATTTGCCTTATGATATATGCACTCAGACCCCAACCTAAGTAAATTCTTTAAATACCTTTTATTGTTCTTAATCACAATACTAATCCTCATTACAGCTAATAACCTATTCCAACTATTCATCGGTTGAGAAGGTGTAGGAATTATATCATTCCTCCTCATTGGCTGATGATTCAGCCGAACCGATGCAAACACAGCTGCTCTCCAAGCCGTTATCTATAACCGTATCGGAGATATCGGTTTAATCATAGCCATAGCATGACTAGCCATAAACCTAAACTCATGGGAGATACAACAACTCTTCTTTCTCTCCAAAAACACAAATCTAACCCTTCCGCTTCTAGGGTTAGTTCTAGCAGCAGCTGGAAAATCAGCCCAATTCGGCCTACACCCATGACTTCCAGCCGCCATAGAGGGTCCTACGCCAGTCTCTGCCCTACTTCACTCAAGCACAATAGTAGTTGCAGGTGTCTTCCTACTTATCCGCCTCCACCCTTTAATTCAAGACAACCATGTAATTCTATCAACCTGCCTATGTCTAGGAGCACTTACAACCCTCTTCACAGCTACCTGTGCACTCACCCAAAACGACATCAAAAAAATTATCGCTTTCTCTACATCTAGCCAACTAGGTCTTATAATAGTCACTATTGGTCTCAACCAACCACAGTTGGCCTTCCTCCACATCTGCACACACGCCTTCTTCAAAGCAATGCTCTTCCTATGCTCTGGCTCCATCATCCACAGCCTAAATGATGAACAAGATATCCGAAAAATAGGGGGC

MB05 KR703226.1 TTTTCCTAATCCTTTTATACCCTCTAATCTCATCCATACTTCCCACCAAAACATCACTTAACCACATTTGATCCTCCTCAGTCCATGTTAAAACAGCTGTAAAAACCTCTTTCTTTATTAGCCTTATCCCCTTATTTATCTTCTTAGACCAAGGTTTAGAATCCATCACAACCAACTGAAATTGATTCAACTTAGGGACCCTCGACATTAACATAAGTTTCAAATTTGACACCTATTCCATTATCTTCACTCCCGTAGCCCTATATGTAACCTGATCTATCCTAGAATTTGCCTTATGATATATGCACTCAGACCCCAACCTAAGTAAATTCTTTAAATACCTTTTATTGTTCTTAATCACAATACTAATCCTCATTACAGCTAATAACCTATTCCAACTATTCATCGGTTGAGAGGGTGTAGGAATTATATCATTCCTCCTCATTGGCTGATGACTCAGCCGAACCGATGCAAACACAGCTGCTCTCCAAGCCGTTATCTATAACCGTATCGGAGATATCGGTTTAATCATAGCCATAGCATGACTAGCCATAAACCTAAACTCATGGGAGATACAACAACTCTTCTTTCTCTCCAAAAACACAAATCTAACCCTTCCGCTTCTAGGGTTAGTTCTAGCAGCAGCTGGAAAATCAGCCCAATTCGGCCTACACCCATGACTTCCAGCCGCCATAGAGGGTCCTACGCCAGTCTCTGCCCTACTTCACTCAAGCACAATAGTAGTTGCAGGTGTCTTCCTACTTATCCGCCTCCACCCTTTAATTCAAGACAACCATGTAATTCTATCAACCTGCCTATGTCTAGGGGCACTTACAACCCTCTTCACAGCTACCTGTGCACTCACCCAAAACGACATCAAAAAAATTATCGCTTTCTCTACATCTAGCCAACTAGGTCTTATAATAGTCACTATTGGTCTCAACCAACCACAGTTGGCCTTCCTCCACATCTGCACACACGCCTTCTTCAAAGCAATGCTCTTCCTATGCTCTGGCTCCATCATCCACAGCCTAAATGATGAACAAGATATCCGAAAAATAGGGGGC

MB08 KR703229.1 TTTTCCTAATCCTTTTATACCCTCTAATCTCATCCATACTTCCCACCAAAACATCACTTAACCACATCTGATCCTCCTCAGTCCATGTTAAAACAGCTGTAAAAACCTCTTTCTTTATTAGCCTTATCCCCTTATTTATCTTCTTAGACCAAGGTTTAGAATCCATCACAACCAACTGAAATTGATTCAACTTAGGGACCCTCGACATTAACATAAGTTTCAAATTTGACACCTATTCCATTATCTTCACTCCCGTAGCCCTATATGTAACCTGATCTATCCTAGAATTTGCCTTATGATATATGCACTCAGACCCCAACCTAAGTAAATTCTTTAAATACCTTTTATTGTTCTTAATCACAATACTAATCCTCATTACAGCTAATAACCTATTCCAACTATTCATCGGTTGAGAAGGTGTAGGAATTATATCATTCCTCCTCATTGGCTGATGATTCAGCCGAACCGATGCAAACACAGCTGCTCTCCAAGCCGTTATCTACAACCGTATCGGAGATATCGGTTTAATCATAGCCATAGCATGACTAGCCATAAACCTAAACTCATGGGAGATACAACAACTCTTCTTTCTCTCCAAAAACACAAATCTAACCCTTCCGCTTCTAGGGTTAGTTCTAGCAGCAGCTGGAAAATCAGCCCAATTCGGCCTACACCCATGACTTCCAGCCGCCATAGAGGGTCCTACGCCAGTCTCTGCCCTACTTCACTCAAGCACAATAGTAGTTGCAGGTGTCTTCCTACTTATCCGCCTCCACCCTTTAATTCAAGACAACCATGTAATTCTATCAACCTGCCTATGTCTAGGGGCACTTACAACCCTCTTCACAGCTACCTGTGCACTCACCCAAAACGACATCAAAAAAATTATCGCTTTCTCTACATCTAGCCAACTAGGTCTTATAATAGTCACTATTGGTCTCAACCAACCACAGTTGGCCTTCCTCCACATCTGCACACACGCCTTCTTCAAAGCAATGCTCTTCCTATGCTCTGGCTCCATCATCCACAGCCTAAATGATGAACAAGATATCCGAAAAATAGGGGGC

MB10 KR703231.1 TTTTCCTAATCCTTTTATACCCTCTAATCTCATCCATACTTCCCACCAAAACATCACTTAACCACATTTGATCCTACTCAGTCCATGTTAAAACAGCTGTAAAAACCTCTTTCTTTATTAGCCTTATCCCCTTATTTATCTTCTTAGACCAAGGTTTAGAATCCATCACAACCAACTGAAATTGATTCAACTTAGGGACCCTCGACATTAACATAAGTTTCAAATTTGACACCTATTCCATTATCTTCACTCCCGTAGCCCTATATGTAACCTGATCTATCCTAGAATTTGCCTTATGATATATGCACTCAGACCCCAACCTAAGTAAATTCTTTAAATACCTTTTATTGTTCTTAATCACAATACTAATCCTCATTACAGCTAATAACCTATTCCAACTATTCATCGGTTGAGAAGGTGTAGGAATTATATCATTCCTCCTCATTGGCTGATGATTCAGCCGAACCGATGCAAACACAGCTGCTCTCCAAGCCGTTATCTATAACCGTATCGGAGATATCGGTTTAATCATAGCCATAGCATGACTAGCCATAAACCTAAACTCATGGGAGATACAACAACTCTTCTTTCTCTCCAAAAACACAAATCTAACCCTTCCGCTTCTAGGGTTAGTTCTAGCAGCAGCTGGAAAATCAGCCCAATTCGGCCTACACCCATGACTTCCAGCCGCCATAGAGGGTCCTACGCCAGTCTCTGCCCTACTTCACTCAAGCACAATAGTAGTTGCAGGTGTCTTCCTACTTATCCGCCTCCACCCTTTAATTCAAGACAACCATGTAATTCTATCAACCTGCCTATGTCTAGGGGCACTTACAACCCTCTTCACAGCTACCTGTGCACTCACCCAAAACGACATCAAAAAAATTATCGCTTTCTCTACATCTAGCCAACTAGGTCTTATAATAGTCACTATTGGTCTCAACCAACCACAGTTGGCCTTCCTCCACATCTGCACACACGCCTTCTTCAAAGCAATGCTCTTCCTATGCTCTGGCTCCATCATCCACAGCCTAAATGATGAACAAGATATCCGAAAAATAGGGGGC

MB12 KR703233.1 TTTTCCTAATCCTTTTATACCCTCTAATCTCATCCATACTTCCCACCAAAACATCACTTAACCACATTTGATCCTCCTCAGTCCATGTCAAAACAGCTGTAAAAACCTCTTTCTTTATTAGCCTTATCCCCTTATTTATCTTCTTAGACCAAGGTTTAGAATCCATCACAACCAACTGAAATTGGTTCAACTTAGGGACCCTCGACATTAACATAAGTTTCAAATTTGACACCTATTCCATTATCTTCACTCCCGTAGCCCTATATGTAACCTGGTCTATCCTAGAATTTGCCTTATGATATATGCACTCAGACCCCAACCTAAATAAATTCTTTAAATACCTTTTATTGTTCTTAATCACAATACTAATCCTCATTACAGCTAATAACCTATTCCAACTATTCATCGGTTGAGAAGGTGTAGGAATTATATCATTCCTCCTCATTGGCTGATGATTCAGCCGAACCGATGCAAACACAGCTGCTCTCCAAGCCGTTATCTATAACCGTATCGGAGATATCGGTTTAATCATAGCCATAGCATGACTAGCCATAAACCTAAACTCATGAGAGATACAACAACTCTTCTTTCTCTCCAAAAACACAAATCTAACCCTTCCACTTCTAGGGTTAGTTCTAGCAGCAGCTGGAAAATCAGCCCAATTCGGCCTACACCCATGACTTCCAGCCGCCATAGAGGGTCCTACGCCAGTCTCTGCCCTACTTCACTCAAGCACAATAGTAGTTGCAGGTGTCTTCCTACTTATCCGCCTCCACCCTTTAATTCAAGACAACCATGTAATTCTATCAACCTGTCTATGTCTAGGGGCACTTACAACCCTCTTCACAGCTACCTGTGCACTCACCCAGAACGACATCAAAAAAATTATCGCTTTCTCTACATCTAGCCAACTAGGTCTTATAATAGTCACTATTGGTCTCAACCAACCACAGTTGGCCTTCCTCCACATCTGCACACACGCCTTCTTCAAAGCAATGCTCTTCCTATGCTCTGGCTCCATCATCCACAGCCTAAATGATGAACAAGATATCCGAAAAATAGGGGGC

MB02 KR703223.1 TTTTCCTGATCCTTTTATACCCTCTAATCTCATCCATACTTCCCACCAAAACATCACTTAACCACATTTGATCCTCCTCAGTCCATGTTAAAACAGCTGTAAAAACCTCTTTCTTTATTAGCCTTATCCCCTTATTTATCTTCTTAGACCAAGGTTTAGAATCCATCACAACCAACTGAAATTGATTCAACTTAGGGACCCTCGACATTAACATAAGTTTCAAATTTGACACCTATTCCATTATCTTCACTCCCGTAGCCCTATATGTAACCTGATCTATCCTAGAATTTGCCTTATGATATATGCACTCAGACCCCAACCTAAGTAAATTCTTTAAATACCTTTTATTGTTCTTAATCACAATACTAATCCTCATTACAGCTAATAACCTATTCCAACTATTCATCGGTTGAGAAGGTGTAGGAATTATATCATTCCTCCTCATTGGCTGATGATTCAGCCGAACCGATGCAAACACAGCTGCTCTCCAAGCCGTTATCTATAACCGTATCGGAGATATCGGTTTAATCATAGCCATAGCATGACTAGCCATAAACCTAAACTCATGGGAGATACAACAACTCTTCTTTCTCTCCAAAAACACAAATCTAACCCTTCCGCTTCTAGGGTTAGTTCTAGCAGCAGCTGGAAAATCAGCCCAATTCGGCCTACACCCATGACTTCCAGCCGCCATAGAGGGTCCTACGCCAGTCTCTGCCCTACTTCACTCAAGCACAATAGTAGTTGCAGGTGTCTTCCTACTTATCCGCCTCCACCCTTTAATTCAAGACAACCATGTAATTCTATCAACCTGCCTATGTCTAGGGGCACTTACAACCCTCTTCACAGCTACCTGTGCACTCACCCAAAACGACATCAAAAAAATTATCGCTTTCTCTACATCTAGCCAACTAGGTCTTATAATAGTCACTATTGGTCTCAACCAACCACAGTTGGCCTTCCTCCACATCTGCACACACGCCTTCTTCAAAGCAATGCTCTTCCTATGCTCTGGCTCCATCATCCACAGCCTAAATGATGAACAAGATATCCGAAAAATAGGGGGC

MB01 KR703222.1 TTTTCCTAATCCTTTTATACCCTCTAATCTCATCCATACTTCCCACCAAAACATCACTTAACCACATTTGATCCTCCTCAGTCCATGTTAAAACAGCTGTAAAAACCTCTTTCTTTATTAGCCTTATCCCCTTATTTATCTTCTTAGACCAAGGTTTAGAATCCATCACAACCAACTGAAATTGATTCAACTTAGGGACCCTCGACATTAACATAAGTTTCAAATTTGACACCTATTCCATTATCTTCACTCCCGTAGCCCTATATGTAACCTGATCTATCCTAGAATTTGCCTTATGATATATGCACTCAGACCCCAACCTAAGTAAATTCTTTAAATACCTTTTATTGTTCTTAATCACAATACTAATCCTCATTACAGCTAATAACCTATTCCAACTATTCATCGGTTGAGAAGGTGTAGGAATTATATCATTCCTCCTCATTGGCTGATGATTCAGCCGAACCGATGCAAACACAGCTGCTCTCCAAGCCGTTATCTATAACCGTATCGGAGATATCGGTTTAATCATAGCCATAGCATGACTAGCCATAAACCTAAACTCATGGGAGATACAACAACTCTTCTTTCTCTCCAAAAACACAAATCTAACCCTTCCGCTTCTAGGGTTAGTTCTAGCAGCAGCTGGAAAATCAGCCCAATTCGGCCTACACCCATGACTTCCAGCCGCCATAGAGGGTCCTACGCCAGTCTCTGCCCTACTTCACTCAAGCACAATAGTAGTTGCAGGTGTCTTCCTACTTATCCGCCTCCACCCTTTAATTCAAGACAACCATGTAATTCTATCAACCTGCCTATGTCTAGGGGCACTTACAACCCTCTTCACAGCTACCTGTGCACTCACCCAAAACGACATCAAAAAAATTATCGCTTTCTCTACATCTAGCCAACTAGGTCTTATAATAGTCACTATTGGTCTCAACCAACCACAGTTGGCCTTCCTCCACATCTGCACACACGCCTTCTTCAAAGCAATGCTCTTCCTATGCTCTGGCTCCATCATCCACAGCCTAAATGATGAACAAGATATCCGAAAAATAGGGGGC

MB03 KR703224.1 TTTTCCTAATCCTTTTATACCCTCTAATCTCATCCATACTTCCCACCAAAACATCACTTAACCACATTTGATCCTCCTCAGTCCATGTTAAAACAGCTGTAAAAACCTCTTTCTTTATTAGCCTTATCCCCTTATTTATCTTCTTAGACCAAGGTTTAGAATCCATCACAACCAACTGAAATTGATTCAACTTAGGGACCCTCGACATTAACATGAGTTTCAAATTTGACACCTATTCCATTATCTTCACTCCCGTAGCCCTATATGTAACCTGATCTATCCTAGAATTTGCCTTATGATATATGCACTCAGACCCCAACCTAAGTAAATTCTTTAAATACCTTTTATTGTTCTTAATCACAATACTAATCCTCATTACAGCTAATAACCTATTCCAACTATTCATCGGTTGAGAAGGTGTAGGAATTATATCATTCCTCCTCATTGGCTGATGATTCAGCCGAACCGATGCAAACACAGCTGCTCTCCAAGCCGTTATCTATAACCGTATCGGAGATATCGGTTTAATCATAGCCATAGCATGACTAGCCATAAACCTAAACTCATGGGAGATACAACAACTCTTCTTTCTCTCCAAAAACACAAATCTAACCCTTCCGCTTCTAGGGTTAGTTCTAGCAGCAGCTGGAAAATCAGCCCAATTCGGCCTACACCCATGACTTCCAGCCGCCATAGAGGGTCCTACGCCAGTCTCTGCCCTACTTCACTCAAGCACAATAGTAGTTGCAGGTGTCTTCCTACTTATCCGCCTCCACCCTTTAATTCAAGACAACCATGTAATTCTATCAACCTGCCTATGTCTAGGGGCACTTACAACCCTCTTCACAGCTACCTGTGCACTCACCCAAAACGACATCAAAAAAATTATCGCTTTCTCTACATCTAGCCAACTAGGTCTTATAATAGTCACTATTGGTCTCAACCAACCACAGTTGGCCTTCCTCCACATCTGCACACACGCCTTCTTCAAAGCAATGCTCTTCCTATGCTCTGGCTCCATCATCCACAGCCTAAATGATGAACAAGATATCCGAAAAATAGGGGGC

MAH4 KR703220.1 TTTTCCTAATCCTTTTATACCCTCTAATCTCATCCATACTTCCCACCAAAACATCACTTAACCACATTTGATCCTCCTCAGTCCATGTTAAAACAGCTGTAAAAACCTCTTTCTTTATTAGCCTTATCCCCTTATTTATCTTCTTAGACCAAGGTTTAGAATCCATCACAACCAACTGAAATTGATTCAACTTAGGGACCCTCGACATTAACATAAGTTTCAAATTTGACACCTATTCCATTATCTTCACTCCCGTAGCCCTATATGTAACCTGATCTATCCTAGAATTTGCCTTATGATATATGCACTCAGACCCCAACCTAAGTAAATTCTTTAAATACCTTTTATTGTTCTTAATCACAATACTAATCCTCATTACAGCTAATAACCTATTCCAACTATTCATCGGTTGAGAAGGTGTAGGAATTATATCATTCCTCCTCATTGGCTGATGATTCAGCCGAACCGATGCAAACACAGCTGCTCTCCAAGCCGTTATCTATAACCGTATCGGAGATATCGGTTTAATCATAGCCATAGCATGACTAGCCATAAACCTAAACTCATGGGAGATACAACAACTCTTCTTTCTCTCCAAAAACACAAATCTAACCCTTCCGCTTCTAGGGTTAGTTCTAGCAGCAGCTGGAAAATCAGCCCAATTCGGCCTACACCCATGACTTCCAGCCGCCATAGAGGGTCCTACGCCAGTCTCTGCCCTACTTCACTCAAGCACAATAGTAGTTGCAGGTGTCTTCCTACTTATCCGCCTCCACCCTTTAATTCAAGACAACCATGTAATTCTATCAACCTGCCTATGTCTAGGGGCACTTACAACCCTCTTCACAGCTACCTGTGCACTCACCCAAAACGACATCAAAAAAATTATCGCTTTCTCTACATCTAGCCAACTAGGTCTTATAATAGTCACTATTGGTCTCAACCAACCACAGTTGGCCTTCCTCCACATCTGCACACACGCCTTCTTCAAAGCAATGCTCTTCCTATGCTCTGGCTCCATCATCCACAGCCTAAATGATGAACAAGATATCCGAAAAATAGGGGGC

MB09 KR703230.1 TTTTCCTAATCCTTTTATACCCTCTAATCTCATCCATACTTCCCACCAAAACATCACTTAACCACATTTGATCCTCCTCAGTCCATGTTAAAACAGCTGTAAAAACCTCTTTCTTTATTAGCCTTATCCCCTTATTTATCTTCTTAGACCAAGGTTTAGAATCCATCACAACCAACTGAAATTGATTCAACTTAGGGACCCTCGACATTAACATAAGTTTCAAATTTGACACCTATTCCATTATCTTCACTCCCGTAGCCCTATATGTAACCTGATCTATCCTAGAATTTGCCTTATGATATATGCACTCAGACCCCAACCTAAGTAAATTCTTTAAATACCTTTTATTGTTCTTAATCACAATACTAATCCTCATTACAGCTAATAACCTATTCCAACTATTCATCGGTTGAGAAGGTGTAGGAATTATATCATTCCTCCTCATTGGCTGATGATTCAGCCGAACCGATGCAAACACAGCTGCTCTCCAAGCCGTTATCTATAACCGTATCGGAGATATCGGTTTAATCATAGCCATAGCATGACTAGCCATAAACCTAAACTCATGGGAGATACAACAACTCTTCTTTCTCTCCAAAAACACAAACCTAACCCTTCCGCTTCTAGGGTTAGTTCTAGCAGCAGCTGGAAAATCAGCCCAATTCGGCCTACACCCATGACTTCCAGCCGCCATAGAGGGTCCTACGCCAGTCTCTGCCCTACTTCACTCAAGCACAATAGTAGTTGCAGGTGTCTTCCTACTTATCCGCCTCCACCCTTTAATTCAAGACAACCATGTAATTCTATCAACCTGCCTATGTCTAGGGGCACTTACAACCCTCTTCACAGCTACCTGTGCACTCACCCAAAACGACATCAAAAAAATTATCGCTTTCTCTACATCTAGCCAACTAGGTCTTATAATAGTCACTATTGGTCTCAACCAACCACAATTGGCCTTCCTCCACATCTGCACACACGCCTTCTTCAAAGCAATGCTCTTCCTATGCTCTGGCTCCATCATCCACAGCCTAAATGATGAACAAGATATCCGAAAAATAGGGGGC

MAH2 KR703218.1 TTTTCCTAATCCTTTTATATCCTCTAATCTCATCCATACTTCCCACCAAAACATCACTTAACCACATTTGATCCTCCTCAGTCCATGTCAAAACAGCTGTAAAAACCTCTTTCTTTATTAGCCTTATTCCCTTATTTATCTTCTTAGACCAAGGTTTAGAATCCATCACAACCAACTGAAATTGATTCAACTTAGGGACCCTCGACATTAACATAAGTTTCAAATTTGACACCTATTCCATTATCTTCACTCCCGTAGCCCTATATGTAACCTGATCTATCCTAGAATTTGCCTTATGATATATGCACTCAGACCCCAACCTAAGTAAATTCTTTAAATACCTTTTATTGTTCTTAATCACAATACTAATCCTCATTACAGCTAATAACCTATTCCAACTATTCATCGGTTGAGAAGGTGTAGGAATTATATCATTCCTCCTCATTGGCTGATGATTCAGCCGAACCGATGCAAACACAGCTGCTCTCCAAGCCGTTATCTATAACCGTATCGGAGATATCGGTTTAATCATAGCCATAGCATGACTAGCCATAAACCTAAACTCATGGGAGATACAACAACTCTTCTTTCTCTCCAAAAACACAAATCTAACCCTTCCACTTCTAGGGTTAGTTCTAGCAGCAGCTGGAAAATCAGCCCAATTCGGCCTACACCCATGACTTCCAGCCGCCATAGAGGGTCCTACGCCAGTCTCTGCCCTACTTCACTCAAGCACAATAGTAGTTGCAGGTGTCTTCCTACTTATCCGCCTCCACCCTTTAATTCAAGACAACCATGTAATTCTATCAACCTGCCTATGTCTAGGGGCACTTACAACCCTCTTCACAGCTACCTGTGCACTCACCCAGAACGATATCAAAAAAATTATCGCTTTCTCTACATCTAGCCAACTAGGTCTTATAATAGTCACTATTGGTCTCAACCAACCACAATTGGCCTTCCTCCACATCTGCACACACGCCTTCTTCAAAGCAATGCTCTTCCTATGCTCTGGCTCCATCATCCACAGCCTAAATGATGAACAAGATATCCGAAAAATAGGGGGC

YM03 KX291014 TTTTCCTAATCCTTTTATACCCTCTAATCTCATCCATACTTCCCACCAAAACATCACTTAACCACATTTGATCCTCCTCAATCCATGTCAAAACAGCTGTAAAAACCTCTTTCTTTATTAGCCTTATCCCCTTATTTATCTTCTTAGACCAAGGTTTAGAATCCATCACAACCAACTGAAATTGATTCAACTTAGGGACCCTCGACATTAACATAAGTTTCAAATTTGACACCTATTCCATTATCTTCACTCCCGTAGCCCTATATGTAACCTGATCTATCCTAGAATTTGCCTTATGATATATGCACTCAGACCCCAACCTAAATAAATTCTTTAAATACCTTTTATTGTTCTTAATCACAATACTAATCCTCATTACAGCTAATAACCTATTCCAACTATTCATCGGTTGAGAAGGTGTAGGAATTATATCATTCCTCCTCATTGGCTGATGATTCAGCCGAACCGATGCAAACACAGCTGCTCTCCAAGCCGTTATCTATAACCGTATCGGAGATATCGGTTTAATCATAGCCATAGCATGACTAGCCATAAACCTAAACTCATGAGAGATACAACAACTCTTCTTTCTCTCCAAAAACACAAATCTAACCCTTCCGCTTCTAGGGTTAGTTCTAGCAGCAGCTGGAAAATCAGCCCAATTCGGCCTACACCCATGACTTCCAGCCGCCATAGAGGGTCCTACGCCAGTCTCTGCCCTACTTCACTCAAGCACAATAGTAGTTGCAGGTGTCTTCCTACTTATCCGCCTCCACCCTTTAATTCAAGACAACCATGTAATTCTATCAACCTGTCTATGTCTAGGGGCACTTACAACCCTCTTCACAGCTACCTGTGCACTCACCCAGAACGACATCAAAAAAATTATCGCTTTCTCTACATCTAGCCAACTAGGTCTTATAATAGTCACTATTGGTCTCAACCAACCACAGTTGGCCTTCCTCCACATCTGCACACATGCCTTCTTCAAAGCAATGCTCTTCCTATGCTCTGGCTCCATCATCCACAGCCTAAATGATGAACAAGATATCCGAAAAATAGGGGGC

YM04 KX291015 TTTTCCTAATCCTTTTATACCCTCTAATCTCATCCATACTTCCCACCAAAACATCACTTAACCACATTTGATCCTCCTCAATCCATGTCAAAACAGCTGTAAAAACCTCTTTCTTTATTAGCCTTATCCCCTTATTTATCTTCTTAGACCAAGGTTTAGAATCCATCACAACCAACTGAAATTGATTCAACTTAGGGACCCTCGACATTAACATAAGTTTCAAATTTGACACCTATTCCATTATCTTCACTCCCGTAGCCCTATATGTAACCTGATCTATCCTAGAATTTGCCTTATGATATATGCACTCAGACCCCAACCTAAATAAATTCTTTAAATACCTTTTATTGTTCTTAATCACAATACTAATCCTCATTACAGCTAATAACCTATTCCAACTATTCATCGGTTGAGAAGGTGTAGGAATTATATCATTCCTCCTCATTGGCTGATGATTCAGCCGAACCGATGCAAACACAGCTGCTCTCCAAGCCGTTATCTATAACCGTATCGGAGATATCGGTTTAATCATAGCCATAGCATGACTAGCCATAAACCTAAACTCATGAGAGATACAACAACTCTTCTTTCTCTCCAAAAACACAAATCTAACCCTTCCGCTTCTAGGGTTAGTTCTAGCAGCAGCTGGAAAATCAGCCCAATTCGGCCTACACCCATGACTTCCAGCCGCCATAGAGGGTCCTACGCCAGTCTCTGCCCTACTTCACTCAAGCACAATAGTAGTTGCAGGTGTCTTCCTACTTATCCGCCTCCACCCTTTAATTCAAGACAACCATGTAATTCTATCAACCTGTCTATGTCTAGGGGCACTTACAACCCTCTTCACAGCTACCTGTGCACTCACCCAGAACGACATCAAAAAAATTATCGCTTTCTCTACATCTAGCCAACTAGGTCTTATAATAGTCACTATTGGTCTCAACCAACCACAGTTGGCCTTCCTCCACATCTGCACACATGCCTTCTTCAAAGCAATGCTCTTCCTATGCTCTGGCTCCATCATCCACAGCCTAAATGATGAACAAGATATCCGAAAAATAGGGGGC

YM05 KX291016 TTTTCCTAATCCTTTTATACCCTCTAATCTCATCCATACTTCCCACCAAAACATCACTTAACCACATTTGATCCTCCTCAGTCCATGTTAAAACAGCTGTAAAAACCTCTTTCTTTATTAGCCTTATCCCCTTATTTATCTTCTTAGACCAAGGTTTAGAATCCATCACAACCAACTGAAATTGATTCAACTTAGGGACCCTCGACATTAACATAAGTTTCAAATTTGACACCTATTCCATTATCTTCACTCCCGTAGCCCTATATGTAACCTGATCTATCCTAGAATTTGCCTTATGATATATGCACTCAGACCCCAACCTAAGTAAATTCTTTAAATACCTTTTATTGTTCTTAATCACAATACTAATCCTCATTACAGCTAATAACCTATTCCAACTATTCATCGGTTGAGAGGGTGTAGGAATTATATCATTCCTCCTCATTGGCTGATGATTCAGCCGAACCGATGCAAACACAGCTGCTCTCCAAGCCGTTATCTATAACCGTATCGGAGATATCGGTTTAATCATAGCCATAGCATGACTAGCCATAAACCTAAACTCATGGGAGATACAACAACTCTTCTTTCTCTCCAAAAACACAAATCTAACCCTTCCGCTTCTAGGGTTAGTTCTAGCAGCAGCTGGAAAATCAGCCCAATTCGGCCTACACCCATGACTTCCAGCCGCCATAGAGGGTCCTACGCCAGTCTCTGCCCTACTTCACTCAAGCACAATAGTAGTTGCAGGTGTCTTCCTACTTATCCGCCTCCACCCTTTAATTCAAGACAACCATGTAATTCTATCAACCTGCCTATGTCTAGGGGCACTTACAACCCTCTTCACAGCTACCTGTGCACTCACCCAAAACGACATCAAAAAAATTATCGCTTTCTCTACATCTAGCCAACTAGGTCTTATAATAGTCACTATTGGTCTCAACCAACCACAGTTGGCCTTCCTCCACATCTGCACACACGCCTTCTTCAAAGCAATGCTCTTCCTATGCTCTGGCTCCATCATCCACAGCCTAAATGATGAACAAGATATCCGAAAAATAGGGGGC

YM06 X350065 TTTTCCTAATCCTTTTATACCCTCTAATCTCATCCATACTTCCCACCAAAACATCACTTAACCACATTTGATCCTCCTCAGTCCATGTTAAAACAGCTGTAAAAACCTCTTTCTTTATTAGCCTTATCCCCTTATTTATCTTCTTAGACCAAGGTTTAGAATCCATCACAACCAACTGAAATTGATTCAACTTAGGGACCCTCGACATTAACATAAGTTTCAAATTTGACACCTATTCCATTATCTTCACTCCCGTAGCCCTATATGTAACCTGATCTATCCTAGAATTTGCCTTATGATATATGCACTCAGACCCCAACCTAAGTAAATTCTTTAAATACCTTTTATTGTTCTTAATCACAATACTAATCCTCATTACAGCTAATAACCTATTCCAACTATTCATCGGTTGAGAAGGTGTAGGAATTATATCATTCCTCCTCATTGGCTGATGATTCAGCCGAACCGATGCAAACACAGCTGCTCTCCAAGCCGTTATCTATAACCGTATCGGAGATATCGGTTTAATCATAGCCATAGCATGACTAGCCATAAACCTAAACTCATGGGAGATACAACAACTCTTCTTTCTCTCCAAAAACACAAATCTAACCCTTCCGCTTCTAGGGTTAGTTCTAGCAGCAGCTGGAAAATCAGCCCAATTCGGCCTACACCCATGACTTCCAGCCGCCATAGAGGGTCCTACGCCAGTCTCTGCCCTACTTCACTCAAGCACAATAGTAGTTGCAGGTGTCTTCCTACTTATCCGCCTCCACCCTTTAATTCAAGACAACCATGTAATTCTATCAACCTGCCTATGTCTAGGGGCACTTACAACCCTCTTCACAGCTACCTGTGCACTCACCCAAAACGACATCAAAAAAATTATCGCTTTCTCTACATCTAGCCAACTAGGTCTTATAATAGTCACTATTGGTCTCAACCAACCACAGTTGGCCTTCCTCCACATCTGCACACACGCCTTCTTCAAAGCAATGCTCTTCCTATGCTCTGGCTCCATCATCCACAGCCTAAATGATGAACAAGATATCCGAAAAATAGGGGGC

YM07 KX291017 TTTTCCTAATCCTTTTATACCCTCTAATCTCATCCATACTTCCCACCAAAACATCACTTAACCACATTTGATCCTCCTCAATCCATGTCAAAACAGCTGTAAAAACCTCTTTCTTTATTAGCCTTATCCCCTTATTTATCTTCTTAGACCAAGGTTTAGAATCCATCACAACCAACTGAAATTGATTCAACTTAGGGACCCTCGACATTAACATAAGTTTCAAATTTGACACCTATTCCATTATCTTCACTCCCGTAGCCCTATATGTAACCTGATCTATCCTAGAATTTGCCTTATGATATATGCACTCAGACCCCAACCTAAATAAATTCTTTAAATACCTTTTATTGTTCTTAATCACAATACTAATCCTCATTACAGCTAATAACCTATTCCAACTATTCATCGGTTGAGAAGGTGTAGGAATTATATCATTCCTCCTCATTGGCTGATGATTCAGCCGAACCGATGCAAACACAGCTGCTCTCCAAGCCGTTATCTATAACCGTATCGGAGATATCGGTTTAATCATAGCCATAGCATGACTAGCCATAAACCTAAACTCATGAGAGATACAACAACTCTTCTTTCTCTCCAAAAACACAAATCTAACCCTTCCGCTTCTAGGGTTAGTTCTAGCAGCAGCTGGAAAATCAGCCCAATTCGGCCTACACCCATGACTTCCAGCCGCCATAGAGGGTCCTACGCCAGTCTCTGCCCTACTTCACTCAAGCACAATAGTAGTTGCAGGTGTCTTCCTACTTATCCGCCTCCACCCTTTAATTCAAGACAACCATGTAATTCTATCAACCTGTCTATGTCTAGGGGCACTTACAACCCTCTTCACAGCTACCTGTGCACTCACCCAGAACGACATCAAAAAAATTATCGCTTTCTCTACATCTAGCCAACTAGGTCTTATAATAGTCACTATTGGTCTCAACCAACCACAGTTGGCCTTCCTCCACATCTGCACACATGCCTTCTTCAAAGCAATGCTCTTCCTATGCTCTGGCTCCATCATCCACAGCCTAAATGATGAACAAGATATCCGAAAAATAGGGGGC

YM08 KX291018 TTTTCCTAATCCTTTTATACCCTCTAATCTCATCCATACTTCCCACCAAAACATCACTTAACCACATTTGATCCTCCTCAATCCATGTCAAAACAGCTGTAAAAACCTCTTTCTTTATTAGCCTTATCCCCTTATTTATCTTCTTAGACCAAGGTTTAGAATCCATCACAACCAACTGAAATTGATTCAACTTAGGGACCCTCGACATTAACATAAGTTTCAAATTTGACACCTATTCCATTATCTTCACTCCCGTAGCCCTATATGTAACCTGATCTATCCTAGAATTTGCCTTATGATATATGCACTCAGACCCCAACCTAAATAAATTCTTTAAATACCTTTTATTGTTCTTAATCACAATACTAATCCTCATTACAGCTAATAACCTATTCCAACTATTCATCGGTTGAGAAGGTGTAGGAATTATATCATTCCTCCTCATTGGCTGATGATTCAGCCGAACCGATGCAAACACAGCTGCTCTCCAAGCCGTTATCTATAACCGTATCGGAGATATCGGTTTAATCATAGCCATAGCATGACTAGCCATAAACCTAAACTCATGAGAGATACAACAACTCTTCTTTCTCTCCAAAAACACAAATCTAACCCTTCCGCTTCTAGGGTTAGTTCTAGCAGCAGCTGGAAAATCAGCCCAATTCGGCCTACACCCATGACTTCCAGCCGCCATAGAGGGTCCTACGCCAGTCTCTGCCCTACTTCACTCAAGCACAATAGTAGTTGCAGGTGTCTTCCTACTTATCCGCCTCCACCCTTTAATTCAAGACAACCATGTAATTCTATCAACCTGTCTATGTCTAGGGGCACTTACAACCCTCTTCACAGCTACCTGTGCACTCACCCAGAACGACATCAAAAAAATTATCGCTTTCTCTACATCTAGCCAACTAGGTCTTATAATAGTCACTATTGGTCTCAACCAACCACAGTTGGCCTTCCTCCACATCTGCACACATGCCTTCTTCAAAGCAATGCTCTTCCTATGCTCTGGCTCCATCATCCACAGCCTAAATGATGAACAAGATATCCGAAAAATAGGGGGC

YM09  KX350064 TTTTCCTAATCCTTTTATACCCTCTAATCTCATCCATACTTCCCACCAAAACATCACTTAACCACATTTGATCCTCCTCAATCCATGTCAAAACAGCTGTAAAAACCTCTTTCTTTATTAGCCTTATCCCCTTATTTATCTTCTTAGACCAAGGTTTAGAATCCATCACAACCAACTGAAATTGATTCAACTTAGGGACCCTCGACATTAACATAAGTTTCAAATTTGACACCTATTCCATTATCTTCACTCCCGTAGCCCTATATGTAACCTGATCTATCCTAGAATTTGCCTTATGATATATGCACTCAGACCCCAACCTAAATAAATTCTTTAAATACCTTTTATTGTTCTTAATCACAATACTAATCCTCATTACAGCTAATAACCTATTCCAACTATTCATCGGTTGAGAAGGTGTAGGAATTATATCATTCCTCCTCATTGGCTGATGATTCAGCCGAACCGATGCAAACACAGCTGCTCTCCAAGCCGTTATCTATAACCGTATCGGAGATATCGGTTTAATCATAGCCATAGCATGACTAGCCATAAACCTAAACTCATGAGAGATACAACAACTCTTCTTTCTCTCCAAAAACACAAATCTAACCCTTCCGCTTCTAGGGTTAGTTCTAGCAGCAGCTGGAAAATCAGCCCAATTCGGCCTACACCCATGACTTCCAGCCGCCATAGAGGGTCCTACGCCAGTCTCTGCCCTACTTCACTCAAGCACAATAGTAGTTGCAGGTGTCTTCCTACTTATCCGCCTCCACCCTTTAATTCAAGACAACCATGTAATTCTATCAACCTGTCTATGTCTAGGGGCACTTACAACCCTCTTCACAGCTACCTGTGCACTCACCCAAAACGATATCAAAAAAATTATCGCTTTCTCTACATCTAGCCAACTAGGTCTTATAATAGTCACTATTGGTCTCAACCAACCACAGTTGGCCTTCCTCCACATCTGCACACATGCCTTCTTCAAAGCAATGCTCTTCCTATGCTCTGGCTCCATCATCCACAGCCTAAATGATGAACAAGATATCCGAAAAATAGGGGGC

YM10 KX291019 TTTTCCTAATCCTTTTATACCCTCTAATCTCATCCATACTTCCCACCAAAACATCACTTAACCACATTTGATCCTCCTCAGTCCATGTTAAAACAGCTGTAAAAACCTCTTTCTTTATTAGCCTTATCCCCTTATTTATCTTCTTAGACCAAGGTTTAGAATCCATCACAACCAACTGAAATTGATTCAACTTAGGGACCCTCGACATTAACATAAGTTTCAAATTTGACACCTATTCCATTATCTTCACTCCCGTAGCCCTATATGTAACCTGATCTATCCTAGAATTTGCCTTATGATATATGCACTCAGACCCCAACCTAAGTAAATTCTTTAAATACCTTTTATTGTTCTTAATCACAATACTAATCCTCATTACAGCTAATAACCTATTCCAACTATTCATCGGTTGAGAAGGTGTAGGAATTATATCATTCCTCCTCATTGGCTGATGATTCAGCCGAACCGATGCAAACACAGCTGCTCTCCAAGCCGTTATCTATAACCGTATCGGAGATATCGGTTTAATCATAGCCATAGCATGACTAGCCATAAACCTAAACTCATGGGAGATACAACAACTCTTCTTTCTCTCCAAAAACACAAATCTAACCCTTCCGCTTCTAGGGTTAGTTCTAGCAGCAGCTGGAAAATCAGCCCAATTCGGCCTACACCCATGACTTCCAGCCGCCATAGAGGGTCCTACGCCAGTCTCTGCCCTACTTCACTCAAGCACAATAGTAGTTGCAGGTGTCTTCCTACTTATCCGCCTCCACCCTTTAATTCAAGACAACCATGTAATTCTATCAACCTGCCTATGTCTAGGGGCACTTACAACCCTCTTCACAGCTACCTGTGCACTCACCCAAAACGACATCAAAAAAATTATCGCTTTCTCTACATCTAGCCAACTAGGTCTTATAATAGTCACTATTGGTCTCAACCAACCACAGTTGGCCTTCCTCCACATCTGCACACACGCCTTCTTCAAAGCAATGCTCTTCCTATGCTCTGGCTCCATCATCCACAGCCTAAATGATGAACAAGATATCCGAAAAATAGGGGGC

YM11 KX291020 TTTTCCTAATCCTTTTATACCCTCTAATCTCATCCATACTTCCCACCAAAACATCACTTAACCACATTTGATCCTCCTCAGTCCATGTTAAAACAGCTGTAAAAACCTCTTTCTTTATTAGCCTTATCCCCTTATTTATCTTCTTAGACCAAGGTTTAGAATCCATCACAACCAACTGAAATTGATTCAACTTAGGGACCCTCGACATTAACATAAGTTTCAAATTTGACACCTATTCCATTATCTTCACTCCCGTAGCCCTATATGTAACCTGATCTATCCTAGAATTTGCCTTATGATATATGCACTCAGACCCCAACCTAAGTAAATTCTTTAAATACCTTTTATTGTTCTTAATCACAATACTAATCCTCATTACAGCTAATAACCTATTCCAACTATTCATCGGTTGAGAAGGTGTAGGAATTATATCATTCCTCCTCATTGGCTGATGATTCAGCCGAACCGATGCAAACACAGCTGCTCTCCAAGCCGTTATCTATAACCGTATCGGAGATATCGGTTTAATCATAGCCATAGCATGACTAGCCATAAACCTAAACTCATGGGAGATACAACAACTCTTCTTTCTCTCCAAAAACACAAATCTAACCCTTCCGCTTCTAGGGTTAGTTCTAGCAGCAGCTGGAAAATCAGCCCAATTCGGCCTACACCCATGACTTCCAGCCGCCATAGAGGGTCCTACGCCAGTCTCTGCCCTACTTCACTCAAGCACAATAGTAGTTGCAGGTGTCTTCCTACTTATCCGCCTCCACCCTTTAATTCAAGACAACCATGTAATTCTATCAACCTGCCTATGTCTAGGGGCACTTACAACCCTCTTCACAGCTACCTGTGCACTCACCCAAAACGACATCAAAAAAATTATCGCTTTCTCTACATCTAGCCAACTAGGTCTTATAATAGTCACTATTGGTCTCAACCAACCACAGTTGGCCTTCCTCCACATCTGCACACACGCCTTCTTCAAAGCAATGCTCTTCCTATGCTCTGGCTCCATCATCCACAGCCTAAATGATGAACAAGATATCCGAAAAATAGGGGGC

YM12 KX291021 TTTTCCTAATCCTTTTATACCCTCTAATCTCATCCATACTTCCCACCAAAACATCACTTAACCACATTTGATCCTCCTCAATCCATGTCAAAACAGCTGTAAAAACCTCTTTCTTTATTAGCCTTATCCCCTTATTTATCTTCTTAGACCAAGGTTTAGAATCCATCACAACCAACTGAAATTGATTCAACTTAGGGACCCTCGACATTAACATAAGTTTCAAATTTGACACCTATTCCATTATCTTCACTCCCGTAGCCCTATATGTAACCTGATCTATCCTAGAATTTGCCTTATGATATATGCACTCAGACCCCAACCTAAATAAATTCTTTAAATACCTTTTATTGTTCTTAATCACAATACTAATCCTCATTACAGCTAATAACCTATTCCAACTATTCATCGGTTGAGAAGGTGTAGGAATTATATCATTCCTCCTCATTGGCTGATGATTCAGCCGAACCGATGCAAACACAGCTGCTCTCCAAGCCGTTATCTATAACCGTATCGGAGATATCGGTTTAATCATAGCCATAGCATGACTAGCCATAAACCTAAACTCATGAGAGATACAACAACTCTTCTTTCTCTCCAAAAACACAAATCTAACCCTTCCGCTTCTAGGGTTAGTTCTAGCAGCAGCTGGAAAATCAGCCCAATTCGGCCTACACCCATGACTTCCAGCCGCCATAGAGGGTCCTACGCCAGTCTCTGCCCTACTTCACTCAAGCACAATAGTAGTTGCAGGTGTCTTCCTACTTATCCGCCTCCACCCTTTAATTCAAGACAACCATGTAATTCTATCAACCTGTCTATGTCTAGGGGCACTTACAACCCTCTTCACAGCTACCTGTGCACTCACCCAGAACGACATCAAAAAAATTATCGCTTTCTCTACATCTAGCCAACTAGGTCTTATAATAGTCACTATTGGTCTCAACCAACCACAGTTGGCCTTCCTCCACATCTGCACACATGCCTTCTTCAAAGCAATGCTCTTCCTATGCTCTGGCTCCATCATCCACAGCCTAAATGATGAACAAGATATCCGAAAAATAGGGGGC

YM13 KX291022 TTTTCCTAATCCTTTTATACCCTCTAATCTCATCCATACTTCCCACCAAAACATCACTTAACCACATTTGATCCTCCTCAGTCCATGTTAAAACAGCTGTAAAAACCTCTTTCTTTATTAGCCTTATCCCCTTATTTATCTTCTTAGACCAAGGTTTAGAATCCATCACAACCAACTGAAATTGATTCAACTTAGGGACCCTCGACATTAACATAAGTTTCAAATTTGACACCTATTCCATTATCTTCACTCCCGTAGCCCTATATGTAACCTGATCTATCCTAGAATTTGCCTTATGATATATGCACTCAGACCCCAACCTAAATAAATTCTTTAAATACCTTTTATTGTTCTTAATCACAATACTAATCCTCATTACAGCTAATAACCTATTCCAACTATTCATCGGTTGAGAAGGTGTAGGAATTATATCATTCCTCCTCATTGGCTGATGATTCAGCCGAACCGATGCAAACACAGCTGCTCTCCAAGCCGTTATCTATAACCGTATCGGAGATATCGGTTTAATCATAGCCATAGCATGACTAGCCATAAACCTAAACTCATGGGAGATACAACAACTCTTCTTTCTCTCCAAAAACACAAATCTAACCCTTCCGCTTCTAGGGTTAGTTCTAGCAGCAGCTGGAAAATCAGCCCAATTCGGCCTACACCCATGACTTCCAGCCGCCATAGAGGGTCCTACGCCAGTCTCTGCCCTACTTCACTCAAGCACAATAGTAGTTGCAGGTGTCTTCCTACTTATCCGCCTCCACCCTTTAATTCAAGACAACCATGTAATTCTATCAACCTGCCTATGTCTAGGGGCACTTACAACCCTCTTCACAGCTACCTGTGCACTCACCCAAAACGACATCAAAAAAATTATCGCTTTCTCTACATCTAGCCAACTAGGTCTTATAATAGTCACTATTGGTCTCAACCAACCACAGTTGGCCTTCCTCCACATCTGCACACACGCCTTCTTCAAAGCAATGCTCTTCCTATGCTCTGGCTCCATCATCCACAGCCTAAATGATGAACAAGATATCCGAAAAATAGGGGGC

YM14 KX291023 TTTTCCTAATCCTTTTATACCCTCTAATCTCATCCATACTTCCCACCAAAACATCACTTAACCACATTTGATCCTCCTCAATCCATGTCAAAACAGCTGTAAAAACCTCTTTCTTTATTAGCCTTATCCCCTTATTTATCTTCTTAGACCAAGGTTTAGAATCCATCACAACCAACTGAAATTGATTCAACTTAGGGACCCTCGACATTAACATAAGTTTCAAATTTGACACCTATTCCATTATCTTCACTCCCGTAGCCCTATATGTAACCTGATCTATCCTAGAATTTGCCTTATGATATATGCACTCAGACCCCAACCTAAGTAAATTCTTTAAATACCTTTTATTGTTCTTAATCACAATACTAATCCTCATTACAGCTAATAACCTATTCCAACTATTCATCGGTTGAGAAGGTGTAGGAATTATATCATTCCTCCTCATTGGCTGATGATTCAGCCGAACCGATGCAAACACAGCTGCTCTCCAAGCCGTTATCTATAACCGTATCGGAGATATCGGTTTAATCATAGCCATAGCATGACTAGCCATAAACCTAAACTCATGGGAGATACAACAACTCTTCTTTCTCTCCAAAAACACAAATCTAACCCTTCCGATTCTAGGGTTAGTTCTAGCAGCAGCTGGAAAATCAGCCCAATTCGGCCTACACCCATGACTTCCAGCCGCCATAGAGGGTCCTACGCCAGTCTCTGCCCTACTTCACTCAAGCACAATAGTAGTTGCAGGTGTCTTCCTACTTATCCGCCTCCACCCTTTAATTCAAGACAACCATGTAATTCTATCAACCTGCCTATGTCTAGGGGCACTTACAACCCTCTTCACAGCTACCTGTGCACTCACCCAAAACGACATCAAAAAAATTATCGCTTTCTCTACATCTAGCCAACTAGGTCTTATAATAGTCACTATTGGTCTCAACCAACCACAGTTGGCCTTCCTCCACATCTGCACACACGCCTTCTTCAAAGCAATGCTCTTCCTATGCTCTGGCTCCATCATCCACAGCCTAAATGATGAACAAGATATCCGAAAAATAGGGGGC
